# Directed differentiation of human embryonic stem cells into parathyroid cells and establishment of parathyroid organoids

**DOI:** 10.1111/cpr.13634

**Published:** 2024-03-18

**Authors:** Ge Wang, Yaying Du, Xiaoqing Cui, Tao Xu, Hanning Li, Menglu Dong, Wei Li, Yajie Li, Wenjun Cai, Jia Xu, Shuyu Li, Xue Yang, Yonglin Wu, Hong Chen, Xingrui Li

**Affiliations:** ^1^ Department of Thyroid and Breast Surgery, Tongji Hospital, Tongji Medical College Huazhong University of Science and Technology Wuhan China; ^2^ Department of Clinical and Diagnostic Sciences University of Alabama at Birmingham Birmingham Alabama USA; ^3^ Department of Rehabilitation, Tongji Hospital, Tongji Medical College Huazhong University of Science and Technology Wuhan China

## Abstract

Differentiation of human embryonic stem cells (hESCs) into human embryonic stem cells‐derived parathyroid‐like cells (hESC‐PT) has clinical significance in providing new therapies for congenital and acquired parathyroid insufficiency conditions. However, a highly reproducible, well‐documented method for parathyroid differentiation remains unavailable. By imitating the natural process of parathyroid embryonic development, we proposed a new hypothesis about the in vitro differentiation of parathyroid‐like cells. Transcriptome, differentiation marker protein detection and parathyroid hormone (PTH) secretion assays were performed after the completion of differentiation. To optimize the differentiation protocol and further improve the differentiation rate, we designed glial cells missing transcription factor 2 (GCM2) overexpression lentivirus transfection assays and constructed hESCs‐derived parathyroid organoids. The new protocol enabled hESCs to differentiate into hESC‐PT. HESC‐PT cells expressed PTH, GCM2 and CaSR proteins, low extracellular calcium culture could stimulate hESC‐PT cells to secrete PTH. hESC‐PT cells overexpressing GCM2 protein secreted PTH earlier than their counterpart hESC‐PT cells. Compared with the two‐dimensional cell culture environment, hESCs‐derived parathyroid organoids secreted more PTH. Both GCM2 lentiviral transfection and three‐dimensional cultures could make hESC‐PT cells functionally close to human parathyroid cells. Our study demonstrated that hESCs could differentiate into hESC‐PT in vitro, which paves the road for applying the technology to treat hypoparathyroidism and introduces new approaches in the field of regenerative medicine.

## Graphical abstract

An optimized method for differentiating human embryonic stem cells into parathyroid cells in vitro, with satisfactory repeatability and stability. Highly robust parathyroid hormone secretion and coexpression of parathyroid markers in human embryonic stem cells‐derived parathyroid cells. The first construction of human embryonic stem cells‐derived parathyroid organoids.

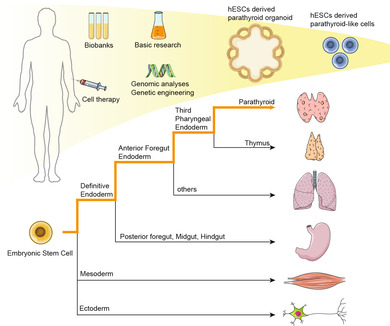

## 1 INTRODUCTION

Parathyroid glands are important endocrine organs, which secrete PTH to regulate calcium homeostasis.^1^ Hypoparathyroidism is an endocrine disorder characterized by low serum calcium levels due to low or absent PTH secretion.^2^ The most common cause of hypoparathyroidism is injuries or accidental removal of parathyroid glands during thyroid surgery, which comprises around 75% of all cases.^3^, ^4^, ^5^ Conventional therapy including calcium supplements, vitamin D supplements and thiazide diuretics only increases the patient's serum calcium concentration but does little for the complications of hypoparathyroidism.^6^, ^7^ The recombinant human parathyroid hormone (rhPTH), as a treatment, was reported to cause osteosarcoma.^8^ Thus, more clinical trials are needed to check the safety of using rhPTH.^9^ Parathyroid allotransplantation is an effective treatment for permanent postoperative hypoparathyroidism but this technique is limited by immune rejection and lack of donors.^10^ Thus, new treatment strategies are needed to treat the hypoparathyroidism‐related problems.

Human pluripotent stem cells exhibit a tremendous potential for regenerative medicine due to their ability to differentiate into different kinds of cell types. There were only two protocols of inducing hESCs to differentiate into hESC‐PT in vitro by sequentially using growth factors and small molecule compounds.^11^, ^12^, ^13^ Bingham's protocol showed that hESCs treated with Activin A and Sonic Hedgehog (Shh) for 26 days could express parathyroid markers and secrete PTH.^11^, ^12^ Another protocol proposed by the Lawton et al. in 2020 mimics the process of parathyroid development, which divides the process of differentiation into multiple stages.^13^ However, no differentiation rate of PTH‐positive cells was clearly reported from those studies.^11^, ^12^, ^13^ Thus, we aimed to develop a new differentiation protocol by mimicking the parathyroids embryonic developmental trajectory.

The lower pair of parathyroid glands and thymus originates from a common primordium formed by the third pharyngeal pouch endoderm. The common primordium splits into dorsal and ventral parts. The dorsal part generates parathyroid glands, while the ventral part leads to thymus.^14^ There are some relatively mature protocols for inducing pluripotent stem cells to differentiate into thymus in vitro.^15^, ^16^ We hypothesized that stem cells first differentiate into the pharyngeal pouch following the differentiation protocols of thymic epithelial cells, and then the usage of small‐molecule compounds activates parathyroid‐related signalling pathways, which can induce cells to differentiate toward the dorsal/proximal region of the pharyngeal pouch, rather than the ventral/distal region, ultimately forming the parathyroid gland. In the present study, we tested the hypothesis and presented a new scheme to direct the differentiation of hESCs to hESC‐PT. The successful differentiation of hESC‐PT not only provides a new therapy option for patients with hypoparathyroidism but also further deepens our understanding of the parathyroid differentiation and development process.

## 2 RESULTS

### 2.1 Exploring a better parathyroid differentiation protocol by mimicking the human embryonic development process

**FIGURE 1:**
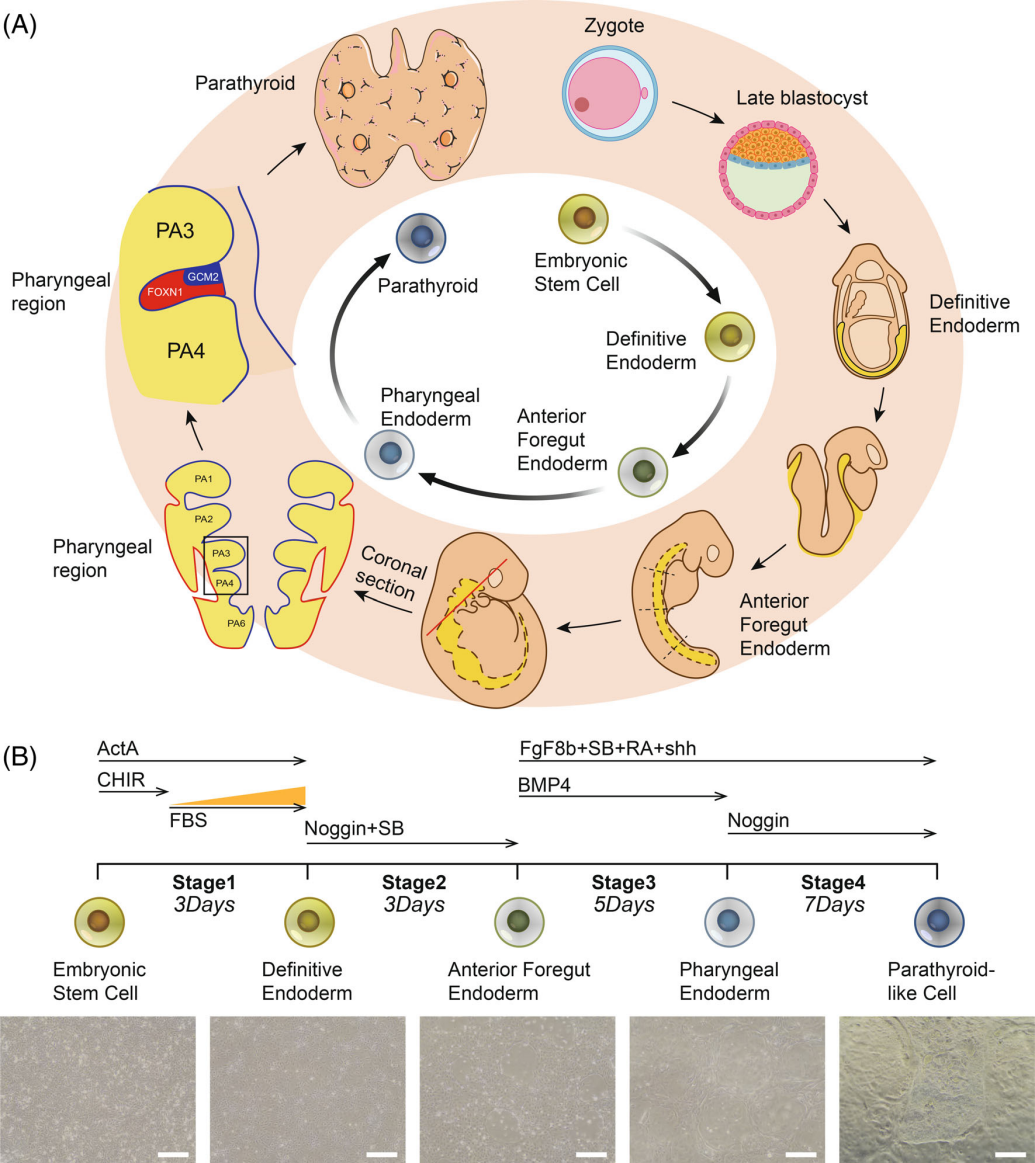
Embryonic development of human parathyroid glands and a new protocol of in vitro parathyroid differentiation. (A) Timeline of parathyroid formation during human embryogenesis. (B) In vitro differentiation scheme that guides human embryonic stem cells into hESC‐PT and the representative white light pictures. ActA, activin A; CHIR, CHIR99021; E, embryonic day; FBS, foetal bovine serum; FgF8b, fibroblast growth factor 8b; PA, pharyngeal arch; RA, retinoic acid; SB, SB431542; Shh, Sonic hedgehog. Scale bar: 200 μm (B).

In order to achieve directed differentiation of embryonic stem cells into hESC‐PT in vitro, it is necessary to understand the human embryonic development process of the parathyroids (Figure 1A). The developmental stage map is based on the developmental process of the parathyroids. As shown in Figure 1B, a protocol for parathyroid induction through embryonic stem cell (ESC), definitive endoderm (DE), anterior foregut endoderm (AFE) and pharyngeal endoderm (PE) is constructed. Differentiation protocols for DE and AFE had been extensively studied.^15^, ^17^, ^18^, ^19^, ^20^, ^21^, ^22^ Activin A has been proved for efficiently inducing pluripotent stem cells to differentiate into DE.^20^, ^23^, ^24^ CHIR99021 was used only on the first day of DE differentiation to activate Wnt signalling pathway and promote cell proliferation.^21^, ^25^ Noggin and SB431542 were used together for inhibiting bone morphogenetic protein (BMP) and transforming growth factor (TGF) signalling pathways to promote DE differentiation into AFE.^15^, ^17^, ^18^, ^26^ To induce pharyngeal endoderm, AFE cells were exposed to five factors (FgF8b, Shh, RA, BMP4 and SB431542) to specifically activate FgF, Shh, RA and BMP signalling and suppress TGF signalling pathway. Sequential activation and suppression of these signalling pathways are essential for normal pharyngeal endoderm development. We investigated how different durations of RA exposure affect the pharyngeal pouch differentiation (Figure S1A), which showed that the longer the RA duration, the higher the HOXA3 expression (Figure S1B,C). Therefore, 5 days of RA exposure was determined in Stage 3. As for the stage 4, Shh in the dorsal region of 3rd pharyngeal pouch endoderm could inhibit FOXN1 expression, which promotes parathyroid development.^27^ At the same time, the BMP signalling pathway inhibitor Noggin was used in the stage 4 to reduce the thymogenesis effect of BMP4 and promote the development of parathyroid glands.^14^, ^27^ Following the differentiation hypothesis in Figure 1B, hESCs were induced to differentiate in vitro. The white light images in Figure 1B showed the typical morphological characteristics of five cell stages during the differentiation process.

### 2.2 Direct differentiation of hESCs to pharyngeal endoderm

**FIGURE 2:**
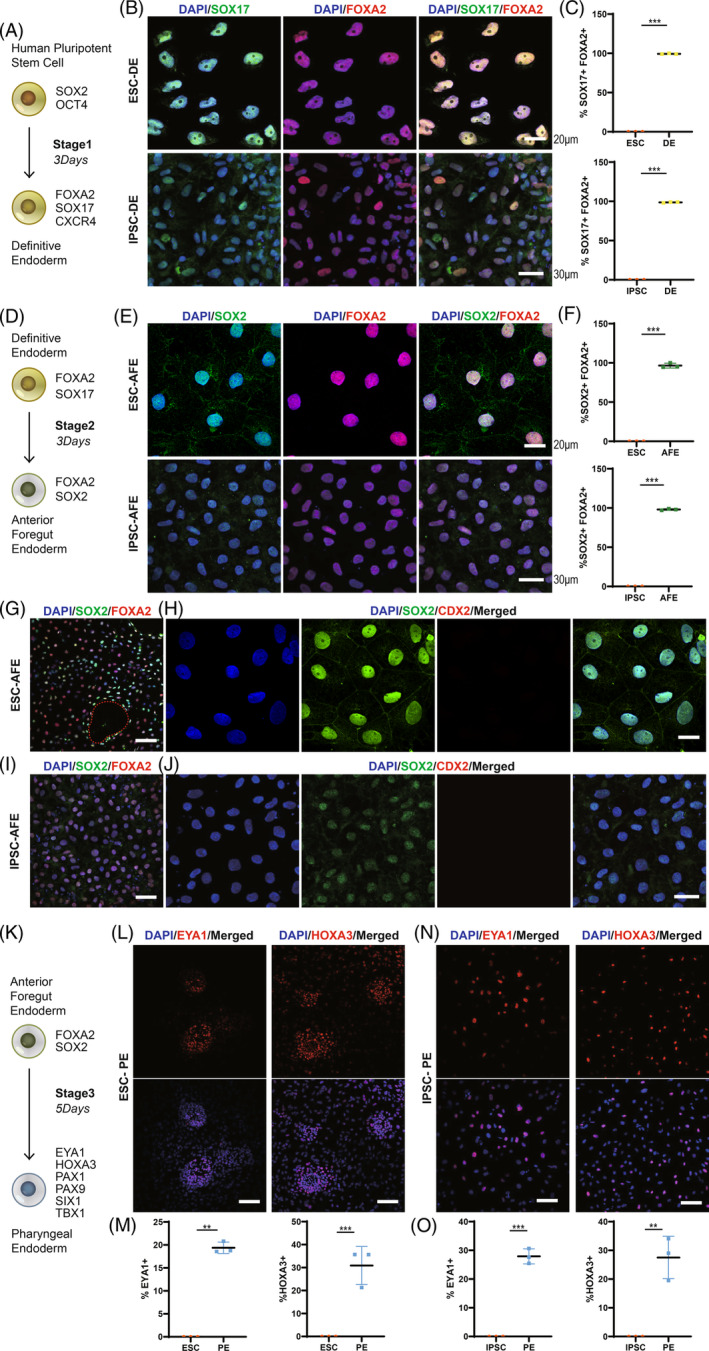
Differentiation of pharyngeal endoderm from human pluripotent stem cells. (A) A schematic diagram of human pluripotent stem cell to definitive endoderm. (B) Co‐immunostaining of SOX17 with FOXA2 in ESC/IPSC‐derived DE cells. (C) Quantification of cell populations that co‐express FOXA2 and SOX17 in ESC/IPSC‐derived DE cells. (D) A differentiation scheme of definitive endoderm to anterior foregut endoderm. (E) Co‐immunostaining of SOX2 with FOXA2 in ESC/IPSC‐derived AFE cells. (F) Quantification of cell populations that co‐express FOXA2 and SOX2 in ESC/IPSC‐derived AFE cells. (G) Typical cavity‐like structure in AFE stage (Red dashed circle). (H) Co‐immunostaining of foregut‐related gene SOX2 and hindgut‐associated gene CDX2 in ESC‐derived AFE cells. (I) Co‐immunostaining of SOX2 with FOXA2 in IPSC‐derived AFE cells. (J) Co‐immunostaining of foregut‐related gene SOX2 and hindgut‐associated gene CDX2 in IPSC‐derived AFE cells. (K) A differentiation scheme of anterior foregut endoderm to pharyngeal endoderm. (L) Co‐immunostaining of EYA1/HOXA3 with DAPI in ESC‐derived PE cells. (M) Quantification of cell populations that express EYA1/HOXA3 in ESC‐derived PE cells. (N) Co‐immunostaining of EYA1/HOXA3 with DAPI in IPSC‐derived PE cells. (O) Quantification of cell populations that express EYA1/HOXA3 in IPSC‐derived PE cells. AFE, anterior foregut endoderm; DE, definitive endoderm; ESC, embryonic stem cell; IPSC, induced pluripotent stem cell; PE, pharyngeal endoderm. Scale bar: 20 μm (H); 30 μm (J); 50 μm (I); 100 μm (G, L and N). Statistics: Data were presented as means ± SD. (C, F, M and O) paired two‐sided t‐test. **p < 0.01, ***p < 0.001.

The differentiation hypothesis was tested by detecting the expression of differentiation markers in the five stages by PCR and immunofluorescence experiments. Meanwhile, another human pluripotent stem cell line, induced pluripotent stem cell (D90D IPSC), was used to validate the reliability of our approach. We first induced human pluripotent stem cells to develop into DE in vitro (Figures 2A and S2A). Immunofluorescence microscopy was used to ascertain the SOX17 and FOXA2 protein expression in hESC/IPSC‐derived DE cells (Figure 2B). Quantitative analysis revealed 99.3% ± 0.3% of hESC‐derived DE cells and 98.7% ± 0.3% of IPSC‐derived DE cells simultaneously expressed SOX17 and FOXA2 (Figures 2C and S6). These results demonstrated a successful differentiation of DE. Noggin and SB431542 were used together for 3 days to induce DE to AFE (Figures 2D and S2B). Immunofluorescence assay was used to ascertain SOX2 and FOXA2 protein expression in hESC/IPSC‐derived AFE cells (Figure 2E). Quantitative analysis showed that 96.5% ± 1.8% of hESC‐derived AFE cells and 98.0% ± 0.6% of IPSC‐derived AFE cells co‐expressed SOX2 and FOXA2 (Figures 2F and S7). Consistent with the above white light pictures (Figure 1B), annular cavity was also seen in the hESC‐derived AFE immunofluorescence image (Figure 2G). This typical morphology was not seen in IPSC‐derived AFE cells, which may be related to cell line difference (Figure 2I). Immunofluorescence data showed that the SOX2 protein (anterior foregut endoderm marker) was positive and the CDX2 protein (posterior foregut endoderm marker, midgut marker and hindgut marker) was negative, which confirmed the anterior foregut endoderm cell type (Figure 2H,J). Negative controls and positive controls for immunofluorescence were shown in Figures S3 and S4A–D. The third stage was to induce differentiation of AFE into the pharyngeal endoderm (Figures 2K and S2C). Immunofluorescence staining of the hESC‐derived PE cells showed both EYA1‐positive cells and HOXA3‐positive cells formed clusters (Figure 2L). Quantitative analysis showed that 19.4% ± 0.7% of cells expressed EYA1 and 30.9% ± 4.8% of cells expressed HOXA3 in the human ESC‐derived PE cells (Figures 2M and S8). The feasibility of the differentiation protocol was also validated by IPSC (Figure 2N). Quantitative analysis showed that 27.9% ± 1.5% of cells expressed EYA1 and 27.5% ± 4.3% of cells expressed HOXA3 in the IPSC‐derived PE cells (Figures 2O and S8). All these findings confirmed a successful differentiation of the pharyngeal endoderm cells.

### 2.3 Direct differentiation of pharyngeal endoderm to hESC‐PT

**FIGURE 3:**
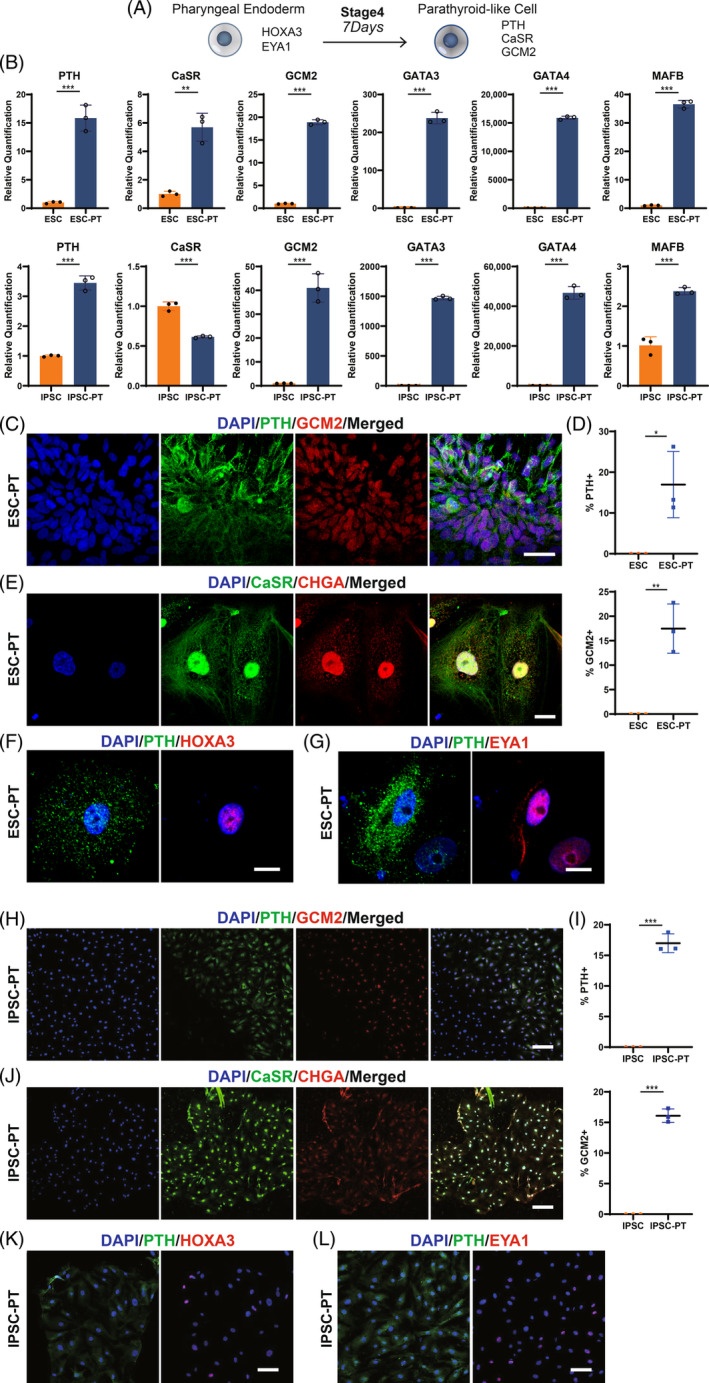
Differentiation of hESC‐PT from pharyngeal endoderm cells. (A) A differentiation scheme of the pharyngeal endoderm cell to the parathyroid‐like cell. (B) RT‐qPCR showing expression of parathyroid‐related transcription factor genes PTH, CaSR, GCM2, GATA3, GATA4, MAFB in hESC, hESC‐PT, IPSC, IPSC‐PT. (C) Co‐immunostaining of PTH with GCM2 in hESC‐PT. (D) Quantification of cell populations that express PTH/GCM2 in hESC‐derived PT cells. (E) Co‐immunostaining of CaSR with CHGA in hESC‐PT. (F) Co‐immunostaining of PTH with HOXA3 in hESC‐PT. (G) Co‐immunostaining of PTH with EYA1 in hESC‐PT. (H) Co‐immunostaining of PTH with GCM2 in IPSC‐PT. (I) Quantification of cell populations that express PTH/GCM2 in IPSC‐derived PT cells. (J) Co‐immunostaining of CaSR with CHGA in IPSC‐PT. (K) Co‐immunostaining of PTH with HOXA3 in IPSC‐PT. (L) Co‐immunostaining of PTH with EYA1 in IPSC‐PT. CaSR, calcium‐sensing receptor; hESC‐PT, embryonic stem cells‐derived parathyroid‐like cells; IPSC‐PT, induced pluripotent stem cells‐derived parathyroid‐like cells. Scale bar: 10 μm (F, G); 20 μm (E); 30 μm (C); 100 μm (K, L); 200 μm (H, J). Statistics: Data are presented as means ± SD. (B, D, I) paired two‐sided t‐test. *p < 0.05, **p < 0.01, ***p < 0.001.

By activating the Shh signalling pathway and inhibiting the BMP signalling pathway, pharyngeal endoderm cells differentiated into hESC‐PT (Figure 3A). Compared with undifferentiated human ESC and IPSC, both hESC‐derived PT and IPSC‐derived PT cells expressed the characteristic PTH mRNA (Figure 3B). Calcium‐sensing receptor (CaSR) is located on the adult parathyroid cells and can sense the change of calcium level in extracellular fluid.^28^, ^29^ GCM2, GATA3, GATA4 and MAFB are key regulatory genes during parathyroid embryonic development. The up‐regulation of these genes corroborated the successful differentiation of hESC‐PT (Figure 3B). Meanwhile, repeatability of the differentiation process and the time course of genes expression at all differentiation stages supported the feasibility of the differentiation protocol (Figure S5). Immunofluorescence assays showed that hESC‐derived PT cells expressed PTH (17.0% ± 4.7%) and GCM2 (17.5% ± 2.9%) proteins (Figures 3C,D and S9). Chromogranin A (CHGA) is an acidic protein found in large dense‐core secretory vesicles, which is involved in the process of PTH secretion. The appearance of CaSR and CHGA proteins suggested that hESC‐PT cells could receive external calcium signal, and then formed secretory vesicles to secrete PTH (Figure 3E). Pharyngeal endoderm marker genes HOXA3 and EYA1 were detected in stage 4 by immunofluorescence (Figure 3F,G). This suggested that HOXA3 and EYA1 play important roles in both the third and fourth stages of parathyroid differentiation. Experiments with another cell line showed similar results compared with hESC (Figure 3H). Quantitative analysis demonstrated that 17.0% ± 0.9% of IPSC‐derived PT cells expressed PTH protein, and 16.1% ± 0.6% of IPSC‐derived PT cells expressed GCM2 protein (Figures 3I and S9). Meanwhile, CaSR, CHGA, HOXA3 and EYA1 proteins were observed in some IPSC‐PT cells (Figure 3J–L).

### 2.4 Bulk RNA sequencing and PTH secretion detection of hESC‐PT

**FIGURE 4:**
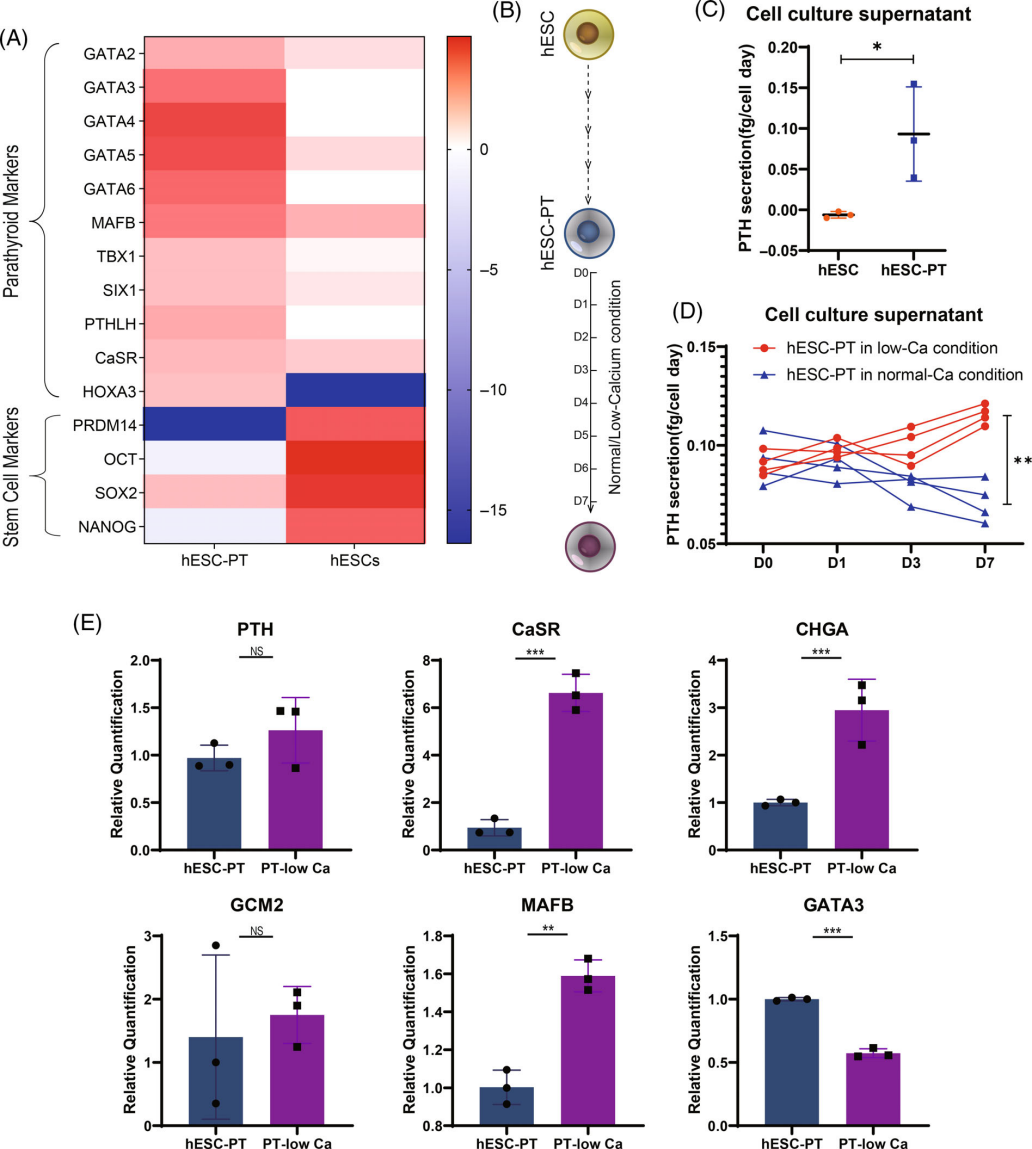
Parathyroid hormone secretion test of hESC‐PT. (A) A heatmap indicating gene expression of parathyroid markers and stem cell markers in hESC‐PT and hESC. (B) Schematic diagram of culturing hESC‐PT in low/normal calcium medium for 7 days. (C) Daily PTH secretion per hESC‐PT cell in cell culture supernatant was detected by ELISA. (D) ELISA was used to detect the effect of low calcium treatment on the PTH secretion. (E) RT‐qPCR showed effects of low‐calcium concentration on the expression of parathyroid‐related transcription factor genes PTH, CaSR, CHGA, GCM2, MAFB, GATA3. hESC‐PT, human embryonic stem cells‐derived parathyroid‐like cells; PTH, parathyroid hormone. Statistics: Data are presented as means ± SD. (C, E) paired two‐sided *t*‐test; (D) two‐way ANOVA with Sidak's multiple comparisons. *p < 0.05, **p < 0.01, ***p < 0.001 and NS, not significant.

Bulk RNA sequencing was used to further characterize hESC‐PT cells. Sequencing results showed that compared with the undifferentiated hESCs, the expression of parathyroid markers (GATA family, MAFB, TBX1, SIX1) was significantly increased in hESC‐PT; by contrast, the expression of stemness genes (PRDM14, OCT, SOX2, NANOG) were significantly decreased (Figure 4A). The sequencing results confirmed the successful differentiation of hESC‐PT cells. Secreting PTH is an important indicator of the success of differentiation and maturation of hESC‐PT cells. After differentiation, hESC‐PT cells were cultured with normal/low calcium for 7 days before measuring the secretion of PTH (Figure 4B). PTH concentrations in cell culture supernatants were determined by ELISA. As shown in Figure 4C, the average PTH secretion per hESC‐PT cell per day was 0.09 fg/cell day. After 7 days' culture with low calcium stimulus, the secreting amount of PTH in the culture supernatant increased (0.12 fg/cell day) (Figure 4D). This indicated that the PTH secretion of hESC‐PT was regulated by the concentration of extracellular calcium ions. In addition, PCR experiments were used to quantify the effect of low calcium culture on the expression of parathyroid marker genes in hESC‐PT cells (Figure 4E). These results suggested low calcium ion concentration promoted the formation and release of intracellular PTH‐secreting vesicles.

### 2.5 Effects of GCM2 protein on parathyroid differentiation

**FIGURE 5:**
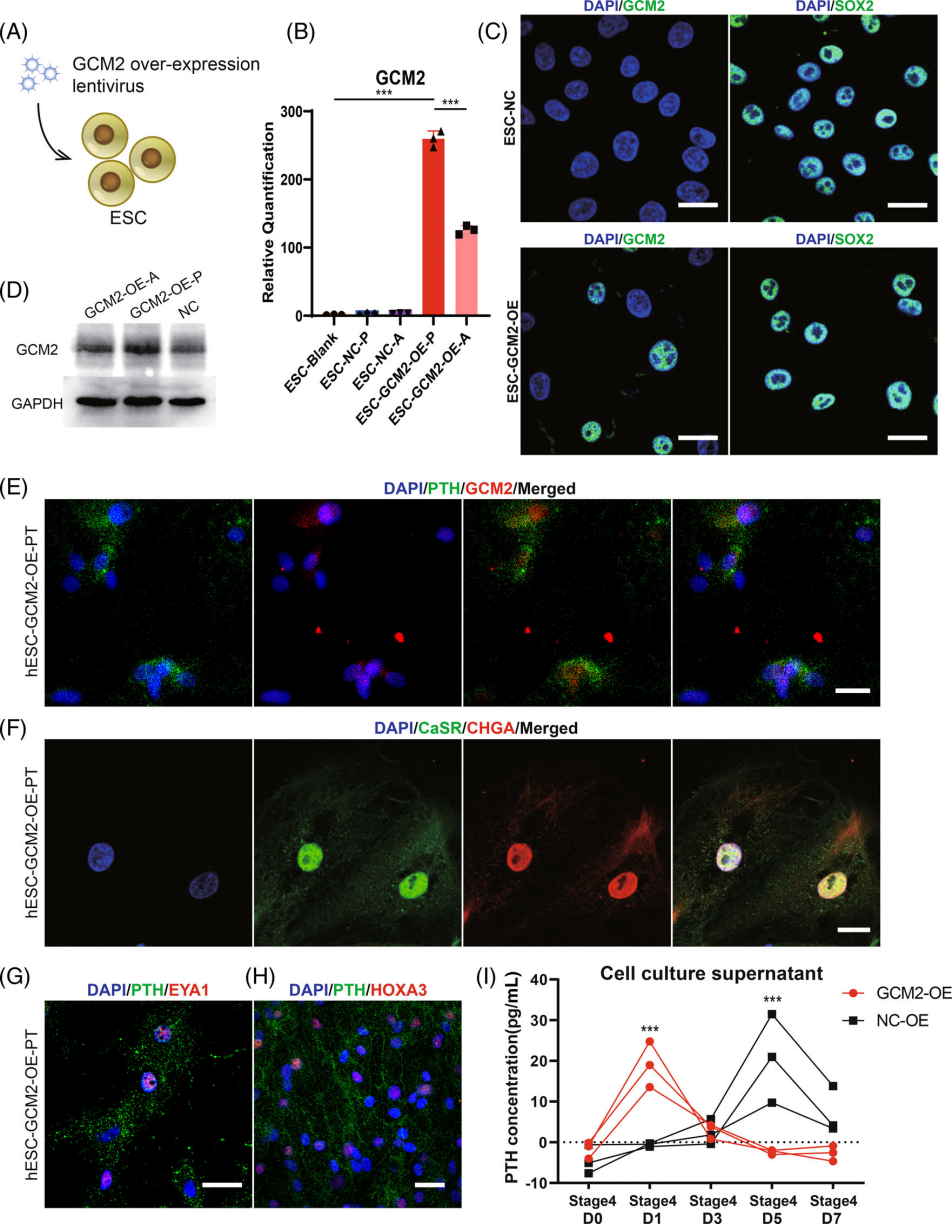
Effects of GCM2 protein overexpression on hESC‐PT cells differentiation. (A) Construction of the H9 cell line that stably overexpressed GCM2 by lentivirus. (B) Overexpression efficiency of GCM2 was validated by RT‐qPCR. ESC‐GCM2‐OE‐A, embryonic stem cells were transfected with GCM2‐overexpression lentivirus by transfection reagent A; ESC‐GCM2‐OE‐P, embryonic stem cells were transfected with GCM2‐overexpression lentivirus by transfection reagent P; ESC‐NC‐A, embryonic stem cells were transfected with negative control lentivirus by transfection reagent A; ESC‐NC‐P, embryonic stem cells were transfected with negative control lentivirus by transfection reagent P. (C) Overexpression efficiency of GCM2 was validated by immunofluorescence. (D) Overexpression efficiency of GCM2 was validated by Western blot. (E) Co‐immunostaining of PTH with GCM2 in GCM2 overexpressed human embryonic stem cells derived parathyroid‐like cells. (F) Co‐immunostaining of CaSR with CHGA in GCM2 overexpressed human embryonic stem cells derived parathyroid‐like cells. (G) Co‐immunostaining of PTH with EYA1 in GCM2 overexpressed human embryonic stem cells derived parathyroid‐like cells. (H) Co‐immunostaining of PTH with HOXA3 in GCM2 overexpressed human embryonic stem cells derived parathyroid‐like cells. (I) ELISA was used to measure the effect of overexpression of GCM2 protein on the secretion of PTH in stage 4. Scale bar: 20 μm (C,E–G); 30 μm (H). Statistics: Data are presented as means ± SD. (B) one‐way ANOVA with Tukey's multiple comparisons; (I) two‐way ANOVA with Sidak's multiple comparisons. ***p < 0.001.

GCM2 is a master gene in parathyroid differentiation. To investigate the function of GCM2 protein in parathyroid embryonic development, hESCs were transfected with GCM2‐overexpressing lentivirus (Figure 5A). Experimental results indicated that GCM2‐overexpressing hESC line was successfully constructed (Figures 5B–D and S10). GCM2‐overexpressing hESCs were then induced to differentiate into hESC‐PT following our induction protocol. As shown in Figure 5E,F, PTH, GCM2, CaSR and CHGA proteins were detected in GCM2‐overexpressing hESC‐PT. However, experimental data showed that overexpression of GCM2 protein did not improve the differentiation rate. HOXA3 and EYA1 protein could also be detected at the end of differentiation by immunofluorescence (Figure 5G,H), which is consistent with the results shown in Figure 3. The ELISA results showed that overexpression of GCM2 protein failed to significantly increase the peak PTH concentration but it took less time to reach the peak PTH secretion (Figure 5I). This suggested that GCM2 protein significantly accelerated cells differentiation.

### 2.6 Establishment of hESC‐derived parathyroid organoids

**FIGURE 6:**
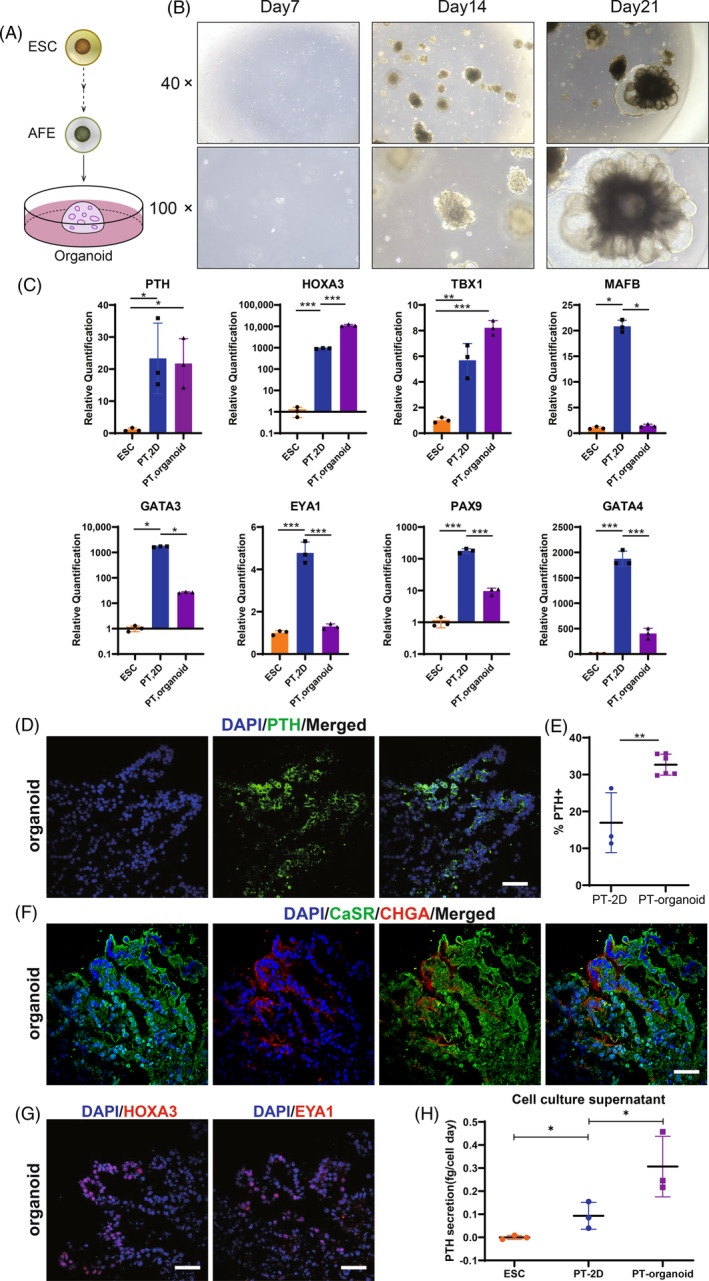
Establishment of hESC‐derived parathyroid organoids. (A) Schematic representation of organoid formation. (B) Representative white light images of the organoids at day 7, 14 and 21. (C) RT‐qPCR showed effects of 3D culture condition on the expression of parathyroid‐related transcription factor genes PTH, HOXA3, Tbx1, MAFB, GATA3, EYA1, PAX9 and GATA4. (D) Co‐immunostaining of PTH with DAPI in hESC‐derived parathyroid organoids. (E) Quantification of cell populations that express PTH in hESC‐derived parathyroid organoids. (F) Co‐immunostaining of CaSR with CHGA in hESC‐derived parathyroid organoids. (G) Immunostaining of HOXA3 and EYA1 in hESC‐derived parathyroid organoids. (H) Daily PTH secretion per hESC‐PT cell in 2D and 3D culture environments were detected by ELISA. Scale bar: 200 μm (B); 50 μm (D, F, G). Statistics: Data are presented as means ± SD. (C, H) one‐way ANOVA with Tukey's multiple comparisons; (E) unpaired two‐sided *t*‐test. *p < 0.05, ***p < 0.001.

The three‐dimensional (3D) parathyroid organoids were constructed to investigate the effects of 3D environment on the parathyroid differentiation of hESCs in vitro. hESCs were differentiated into AFE cells according to the differentiation protocol as seen in Figure 1B, and then AFE cells were mixed with matrigel to construct 3D dome structures (Figure 6A). The white‐light images showed the morphology of hESC‐derived parathyroid organoids at different time points (Figure 6B). The representative structure of parathyroid organoids was a clustered structure composed of multiple hollow acini. The PCR results showed that the expression of HOXA3 was further increased in the 3D culture conditions while the PTH and Tbx1 mRNA levels did not increase compared with the two‐dimensional (2D) culture conditions. However, the expression of MAFB, GATA3, EYA1, PAX9 and GATA4 were all decreased, which may be related to the diverse cell types in organoids (Figure 6C). Representative immunofluorescence staining of frozen organoid sections showed the expression of PTH protein in the hollow acini of organoids, which demonstrated the effectiveness of the protocol for culturing hESC‐derived parathyroid organoids (Figure 6D). Quantitative analysis demonstrated that 32.7% ± 1.2% of hESC‐derived PT organoid cells expressed the PTH (Figures 6E and S11), which indicated organoid cultures could increase the differentiation rate of PTH‐positive cells. Similar to 2D cultures, CaSR and CHGA were detected in hESC‐derived parathyroid organoids (Figure 6F). At the end of parathyroid organoids culture, immunofluorescent staining revealed that some cells in the acini also expressed HOXA3 and EYA1 (Figure 6G). The PTH concentration in the parathyroid organoid culture supernatant was measured by ELISA with an average of 0.25 fg/cell day in the 3D environment (Figure 6H). Our results indicated that hESCs could differentiate into hESC‐PT in the 3D environment in vitro, and the hESC‐derived parathyroid organoids secreted more PTH than the 2D cell cultures.

## 3 DISCUSSION

Physiological and pathological studies of the parathyroid have been limited by the inaccessibility of human parathyroid tissues and the absence of cell lines. Directed differentiation induction of stem cells into parathyroid in vitro can not only generate many cells to accelerate biological and developmental research of the parathyroid but also more importantly can provide a new therapeutic approach for patients with hypoparathyroidism. Unfortunately, the Bingham's protocol cannot be replicated by us possibly due to different cell lines or reagent lots.^11^, ^12^ For the Lawton's protocol, only RNA levels of parathyroid differentiation markers were reported but not protein expression or cell differentiation rates.^13^ In the present study, after comparing the commonalities and differences between parathyroid and thymus embryonic development, the in vitro methods of thymic epithelial cell differentiation were modified to induce the differentiation of hESCs into hESC‐PT.^30^, ^31^ Lentiviral transfection and organoid experiments were performed to explore the role of GCM2 protein and the 3D cell culture environment for the PTH secretion.

The differentiation protocol from AFE to PE has been reported in studies of thymus differentiation.^13^, ^15^, ^16^, ^32^, ^33^ In 2013, Audrey et al. reported the PE differentiation strategy for the first time.^32^ This study reported that a combined use of LY364947, BMP4, RA, Wnt3a, FGF8 and cyclopamine for 2 days could promote the ventral PE cells differentiation. Similar to the Audrey's method, in 2019, Amiet et al. reported a protocol using LY364947, BMP4, EC23, Wnt3a, FGF8b and cyclopamine to differentiate ventral PE cells.^33^ EC23 is a photostable synthetic retinoid with similar chemical actions to ATRA,^34^ which makes the two methods essentially the same.^32^, ^33^ In 2020, Ryo et al. verified a protocol composed of SB431542, BMP4, RA, CHIR99021, FGF8b and cyclopamine to induce PE cells differentiation.^16^ SB431542 is a replacement for LY364947, which is also a selective inhibitor of the TGF‐β type I receptor. CHIR99021 is an activator of the Wnt signalling pathway. Later, they found that withdrawal of BMP4, CHIR99021 or cyclopamine from the induction medium did not affect HOXA3 and TBX1 mRNA expression. The final Ryo's protocol used SB431542, RA and FGF8b in combination for 2 days to differentiate AFE into PE. Also in 2020, Lawton et al. reported a new protocol for in vitro differentiation of parathyroid glands.^13^ Although Lawton et al.'s study only detected the mRNA expression of differentiation markers at each stage, it provided a new way to differentiate the pharyngeal endoderm. Taken together, SB431542, RA and FGF8b are the most used and effective small molecule compounds/cytokines during PE differentiation. RA activates the downstream HOXA3 signalling, which is essential for pharyngeal pouch development.^35^, ^36^, ^37^ In addition, FGF8b was reported for being required for pharyngeal endoderm development.^38^, ^39^

The latest research on differentiating thymic epithelial cells also mentioned the differentiation method of PE. In 2021, Rafael Gras et al. found that the combined use of RA, FGF8b and Shh for 8 days could differentiate AFE into PE.^15^ This is the first use of Shh during PE differentiation, which appears to be paradoxical with cyclopamine (inhibitor of Shh signalling) being used in previous differentiation protocols. Previous embryogenesis studies reported that activation of the Shh signalling pathway was indispensable during pharyngeal pouch and parathyroid development.^27^, ^40^ Shh can activate downstream Tbx1 signalling to promote the formation of pharyngeal endoderm.^41^, ^42^ This is why Shh was used for inducing PE stage in our study. Activation of BMP signalling is necessary during the formation of the pharyngeal pouch as absence of BMP signalling resulted in abnormal parathyroid and thymus morphogenesis.^43^, ^44^ In conclusion, a differentiation protocol including SB431542, RA, FGF8b, Shh and BMP4 was proposed to induce AFE to differentiate into PE in our protocol.

Following the embryonic developmental trajectory of parathyroid glands, cells are induced to differentiate toward the dorsal part of the pharyngeal endoderm after successful PE differentiation. It has been reported that under physiological conditions, Shh signalling in the dorsal region of the third pharyngeal pouch can activate Tbx1, inhibit FOXN1 and limit the development of the thymus.^27^ Meanwhile, Shh can upregulate the expression of GCM2, which ultimately promotes the development of parathyroid glands.^45^ In contrast to the action of Shh, BMP4 signalling in the ventral region of the third pharyngeal endoderm promotes formation and differentiation of thymic epithelial cells.^14^, ^46^ These studies reminded us that Shh should be continued in the fourth stage and BMP4 replaced with Noggin (the BMP4 inhibitor). To summarize, the differentiation method including SB431542, RA, FGF8b, Shh and Noggin was used for 7 days to promote the differentiation of PE into hESC‐PT.

Following our differentiation protocol, hESCs could be differentiated into hESC‐PT. Transcription factors GATA3, GCM2 and MAFB physically interact and form a transcriptional complex, which activates the PTH gene promoter.^47^, ^48^ PCR and Immunofluorescence experiments verified the expressions of parathyroid markers PTH, CaSR, GCM2 and CHGA. It is worth noting that some cells still expressed HOXA3 and EYA1 when they differentiated to the hESC‐PT. This suggests that HOXA3 and EYA1 may play a role in parathyroid differentiation.^36^, ^49^ Compared with undifferentiated hESCs, the daily PTH secretion per hESC‐PT cell reached 0.09 fg/cell day. To our knowledge, this is the first report on the PTH secretion of hESC‐PT differentiated from hESCs in vitro. GCM2 is a key regulator of parathyroid development, which is necessary for the differentiation and survival of parathyroid glands.^50^, ^51^, ^52^ GCM2‐deficient mice failed to develop parathyroid and showed symptoms of hypoparathyroidism.^53^ Meanwhile, GCM2 binds the CaSR promoter and activates the CaSR gene, ultimately promoting the differentiation of the parathyroid glands.^54^ However, only overexpression of GCM2 cannot directly induce hESCs to differentiate into hESC‐PT. Therefore, our differentiation protocol was used to induce GCM2‐overexpressing hESCs into hESC‐PT. Notably, the time to reach peak PTH concentration in GCM2 overexpression group was earlier than their counterpart hESC‐PT cells. An early onset of parathyroid hormone secretion suggested that GCM2 promotes hESC‐PT development and maturation. After reaching the peak secretion, the secretion of PTH gradually decreased. This is consistent with a previous study of parathyroid cells differentiated from tonsil‐derived mesenchymal stem cells.^55^ The gradually decreased secretory function may be due to parathyroid hormone depletion, naive state of hormone‐secreting cells, and unfavourable environment of hESC‐PT cells.

It has been reported that human parathyroid tissues obtained by surgical resection or fine needle aspiration can be coated with matrigel to form patient tissue‐derived parathyroid organoids.^56^ However, to our knowledge, the construction of hESC‐derived parathyroid organoids has never been reported before. We referred many organoids culture methods and coated hESC‐PT cells with matrigel to study the effect of stereoscopic environment on the differentiation and development of hESC‐PT. Immunofluorescence experiments demonstrated the presence of PTH‐positive cells in the organoids. We measured the PTH concentration in the organoid supernatant, which showed that the 3D environment could promote PTH secretion. Consistent with previous studies, the 3D culture environment is more conducive to the maintenance of cell function than the 2D culture.^57^

However, there are several limitations in the current study. For instance, compared with the differentiation rates of stem cell‐derived pancreatic islets and neurons,^58^, ^59^ our differentiation rate of PTH‐positive cells is low (16.96%). In addition, the secretory function of hESC‐PT cells is not stable enough. After reaching the peak secretion, the amount of PTH secretion began to decline. The PTH protein of hESC‐PT/IPSC‐PT cells existed in cytoplasm and nucleus (Figures 3C,H and S9), which was similar to the immunofluorescence staining results of poorly differentiated parathyroid carcinoma cells (Figure S4E). These experimental results also indicated the immaturity of hESC‐PT/IPSC‐PT cells. All these findings would bring difficulties for performing in vivo experiments. Despite the advancements, there are still some gaps in stem cell replacement therapy for patients with hypoparathyroidism. In the future, more in‐depth research is needed to optimize the in vitro differentiation conditions, making it closely resemble to the embryonic development process of parathyroid glands, ultimately improving the low differentiation rate, promoting further maturation of naive cells, and achieving the goal of applying this technology to treat hypoparathyroidism.

## 4 CONCLUSIONS

We successfully developed a modified protocol for inducing hESC into hESC‐PT. hESC‐PT cells secreted PTH, and the amount of PTH secretion is regulated by the extracellular calcium. Overexpression of GCM2 protein accelerated the onset of PTH secretion. The establishment of hESC‐derived parathyroid organoids further increased the percentage of PTH‐positive cells. In short, our study presents a new method for hESC‐PT differentiation in vitro, which lays the foundation for using cell therapy to treat hypoparathyroidism and introduces new approaches in the field of regenerative medicine.

## AUTHOR CONTRIBUTIONS

**Ge Wang:** Conceptualization; investigation; validation; visualization; writing – original draft. **Yaying Du:** Data curation; funding acquisition; validation. **Xiaoqing Cui:** Resources; formal analysis. **Tao Xu:** Software; resources. **Hanning Li:** Visualization; resources. **Menglu Dong:** Formal analysis; methodology. **Wei Li:** Writing – original draft; writing – review and editing. **Yajie Li:** Methodology. **Wenjun Cai:** Writing – original draft. **Jia Xu:** Resources. **Shuyu Li:** Software. **Xue Yang:** Writing – review and editing. **Yonglin Wu:** Writing – review and editing. **Hong Chen:** Conceptualization; data curation; methodology. **Xingrui Li:** Conceptualization; funding acquisition; project administration; supervision.

## CONFLICT OF INTEREST STATEMENT

All the authors have no conflicts of interest to declare.

## Supporting information


**Data S1.** Supporting Information.

**Figure S1.** The effects of different durations of RA exposure on pharyngeal endoderm differentiation. (A) Schematic diagram of different RA action time in each group. (B) Typical white light pictures of each group at the end of pharyngeal endoderm differentiation. (C) RT‐qPCR showed different expression of HOXA3, CDX2, GATA4, GCM2, PAX1, SIX1, TBX1, PAX9, GATA3, EYA1, MAFB and SOX2 in the different durations of RA exposure groups. RA, retinoic acid. Scale bar: 200 μm (B). Statistics: Data are presented as means ± SEM. (C) one‐way ANOVA with Tukey's multiple comparisons. **p < 0.01, ***p < 0.001.

**Figure S2.** Successful expression of the stage I/II/III differentiation markers. (A) RT‐qPCR showed expression of DE‐related transcription factor genes FOXA2, SOX17, MIXL1, CXCR4, NKX2‐1 as well as hESC‐associated genes SOX2. (B) RT‐qPCR showed different expression of FOXA2, SOX17 and SOX2 in ESC/IPSC, DE and AFE stages. (C) RT‐qPCR showing expression of PE‐related transcription factor genes EYA1, HOXA3, PAX1, PAX9, SIX1 and Tbx1. ESC, embryonic stem cell; IPSC, induced pluripotent stem cell; DE, definitive endoderm; AFE, anterior foregut endoderm; PE, pharyngeal endoderm. Statistics: Data are presented as means ± SEM. (A,C) Paired two‐sided *t*‐test; (B) one‐way ANOVA with Tukey's multiple comparisons. *p < 0.05, **p < 0.01, ***p < 0.001.

**Figure S3.** Expression of differentiation markers were measured with immunofluorescence as negative/positive controls. (A) Co‐immunostaining of SOX2 with CDX2 in ESCs. (B) Co‐immunostaining of SOX17 with FOXA2 in ESCs. (C) Co‐immunostaining of PTH with CHGA in ESCs. (D) Co‐immunostaining of CaSR with HOXA3 in ESCs. (E) Immunostaining of EYA1 in ESCs. (F) Immunostaining of Tbx1 in ESCs. (G) Immunostaining of CDX2 in DLD1 cells. (H) Immunostaining of CaSR in DLD1 cells. ESCs, embryonic stem cells. Scale bar: 20 μm (A–F); 30 μm (G,H).

**Figure S4.** For immunofluorescence studies, AFE was used as the negative control while human parathyroid, parathyroid adenoma and parathyroid carcinoma were used as positive controls. (A) Co‐immunostaining of SOX17 with EYA1 in AFE. (B) Immunostaining of HOXA3 in AFE. (C) Co‐immunostaining of PTH, CaSR with CHGA in human parathyroid. (D) Immunostaining of PTH and CaSR in human parathyroid adenoma. (E) The white light image and immunofluorescence staining of primary parathyroid carcinoma cells. AFE, anterior foregut endoderm. Scale bar: 100 μm (C); 20 μm (A, B, E); 10 μm (D).

**Figure S5.** Time course of the differentiation markers at different stages of parathyroid differentiation in vitro. (A) RT‐qPCR showed the time course of differentiation markers in ESC, DE, AFE, PE and PT stages. ESC, embryonic stem cell; DE, definitive endoderm; AFE, anterior foregut endoderm; PE, pharyngeal endoderm; PT, human embryonic stem cells‐derived parathyroid‐like cells. Statistics: Data are presented as means ± SEM. (A) one‐way ANOVA with Tukey's multiple comparisons. *p < 0.05, **p < 0.01, ***p < 0.001.

**Figure S6.** Selection of immunofluorescence visual field and quantification of the percentage of FOXA2‐SOX17 double‐positive cells in the DE stage. (A) Co‐immunostaining of FOXA2 with SOX17 in ESC‐derived DE cells. (B) Co‐immunostaining of FOXA2 with SOX17 in IPSC‐derived DE cells. (C) Co‐immunostaining of FOXA2 with SOX17 in ESC. Scale bar: 100 μm (A); 50 μm (B); 20 μm (C).

**Figure S7.** Selection of immunofluorescence visual field and quantification of the percentage of FOXA2‐SOX2 double‐positive cells in the AFE stage. (A) Co‐immunostaining of FOXA2 with SOX2 in ESC‐derived AFE cells. (B) Co‐immunostaining of FOXA2 with SOX2 in IPSC‐derived AFE cells. Scale bar: 100 μm (A); 50 μm (B).

**Figure S8.** Selection of immunofluorescence visual field and quantification of the percentage of EYA1/HOXA3 positive cells in the PE stage. (A) Immunostaining of EYA1 in ESC‐derived PE cells. (B) Immunostaining of HOXA3 in ESC‐derived PE cells. (C) Immunostaining of EYA1 in IPSC‐derived PE cells. (D) Immunostaining of HOXA3 in IPSC‐derived PE cells. Scale bar: 100 μm (A–D).

**Figure S9.** Selection of immunofluorescence visual field and quantification of the percentage of PTH‐GCM2 double positive cells in the PT stage. (A) Co‐immunostaining of PTH with GCM2 in ESC‐derived PT cells. (B) Co‐immunostaining of PTH with GCM2 in IPSC‐derived PT cells. Scale bar: 100 μm (A); 200 μm (B).

**Figure S10.** Selection of immunofluorescence visual field and quantification of the lentiviral transfection efficiency. (A) RT‐qPCR showed relative quantification of NANOG, OCT4, PRDM14 and SOX2 in ESC‐NC and ESC‐GCM2‐OE cells. (B) Immunostaining of GCM2 in ESC transfected with GCM2‐overexpression lentivirus. (C) Immunostaining of GCM2 in ESC transfected with negative control lentivirus. (D) Quantification of the lentiviral transfection efficiency. Scale bar: 30 μm (A,B). Statistics: Data are presented as means ± SD. (A,D) unpaired two‐sided *t*‐test. ***p < 0.001 and NS, not significant.

**Figure S11.** Selection of immunofluorescence visual field and quantification of the percentage of PTH‐positive cells in the hESC‐derived parathyroid organoids. (A) Immunostaining of PTH in ESC‐derived parathyroid organoids. Scale bar: 100 μm (A).

**Table S1.** Media formulations for stem cell differentiation and organoid culture.
**Table S1A.** The formulation of 500 mL serum‐free basal medium (SFBM).
**Table S1B.** The formulation of definitive endoderm induction media (Day 1 to Day 3).
**Table S1C.** The formulation of anterior foregut endoderm induction media (Day 4 to Day 6).
**Table S1D.** The formulation of pharyngeal endoderm induction media (Day 7 to Day 11).
**Table S1E.** The formulation of parathyroid induction media (Day 12 to Day 18).
**Table S1F.** The formulation of normal calcium concentration parathyroid induction media.
**Table S1G.** The formulation of 500 mL low calcium concentration serum‐free basal medium (SFBM‐Low Ca).
**Table S1H.** The formulation of low calcium concentration parathyroid induction media.
**Table S1I.** The formulation of hESC‐derived parathyroid organoid media.

**Table S2.** Details of the antibody and reagents used in this study.
**Table S2A.** Antibody details and dilution ratios.
**Table S2B.** Details of the reagents used in this study.

**Table S3.** Details of the primers used in this study.

## Data Availability

All data associated with this study are available by contacting the corresponding authors with a reasonable request.
