# Supplementary material for: Directed differentiation of human embryonic stem cells into parathyroid cells and establishment of parathyroid organoids
Source: Cell Prolif. 2024 Mar 18;57(8):e13634. doi: 10.1111/cpr.13634 (PMC11294423; doi:10.1111/cpr.13634)
Supplement: Supplementary file 1 — Data S1. Supporting Information. [file CPR-57-e13634-s008.docx]

1. **Maintenance of hESC and hIPSC**

Human ESC line H9 (WA09 line, NIH registry 0046) and human IPSC line D90D were obtained from WiCell. Culture and expansion of human ESC/IPSC were maintained on feeder-free layers. Corning Matrigel hESC-Qualified Matrix (354277) was used to coat tissue culture-treated cultureware. H9 and D90D were cultured in mTeSR PLUS, and the medium was changed daily. H9 and D90D were passaged by manual scraping to generate cell aggregates, and all the cell lines were used within 50 passages. ESC and IPSC were cultured in a 37° C incubator with 5% CO_2_. Mycoplasma contamination and karyotype were routinely tested.

1. **Induction of Definitive Endoderm**

To increase cell differentiation rate, a single-cell differentiation method was adopted for a better exposure of cells to growth factors/small molecule compounds. Briefly, on day 0, ESC and IPSC were dissociated into single cells and maintained in mTeSR medium with 10 µM Y27632 on Matrigel (354277) coated six-well tissue culture-treated plates. Cells were planted at a density of 2 x 10^5^ cells/cm^2^ so that a 90%-100% confluency could be reached on day 1. On day 1, mTeSR medium was replaced with 1640 supplemented with 100 ng/mL activin A and 3 µM CHIR99021. On day 2, medium was replaced with 1640 supplemented with 100 ng/mL activin A and 0.2% FBS. On day 3, medium was replaced with 1640 supplemented with 100 ng/mL activin A and 2% FBS. On day 4, expression of DE markers of FOXA2 and SOX17 were ascertained by qPCR and immunofluorescence experiments. In this study, the FBS (Thermo Fisher Scientific, cat. no. 16000-044) lots used were 2173495RP, 2330920RP and 1985890, with no significant differences in the differentiation process observed among batches of FBS. Cells that successfully differentiated into DE would proceed to the next stage of differentiation.

1. **Induction of Anterior Foregut Endoderm**

Combined inhibition of BMP and TGF signaling was required to promote the differentiation of DE into AFE. AFE induction media was composed of serum-free basal medium (SFBM) supplemented with 200 ng/mL Noggin and 10 µM SB431542. For the configuration of 500 ml SFBM, 375 ml of IMDM and 125 ml of Ham’s F12 medium were mixed. Then 2.5 ml N2 supplement, 5 ml of B27 supplement, 50 µg/ml of ascorbic acid, 0.45 mM of 1-Thioglycerol (MTG), and 3.33 ml of 7.5% BSA Fraction V were added. SFBM was filtered and protected from light for storage (at 4 °C) for up to 1 month. AFE induction media was changed daily for 3 days (days 4 to 6). AFE markers of FOXA2 and SOX2 were also ascertained by qPCR and immunofluorescence.

1. **Induction of Pharyngeal Endoderm**

Pharyngeal endoderm induction media was composed of SFBM supplemented with 50 ng/ml of FgF8b, 10 μM of SB431542, 0.1 μM of retinoic acid (RA), 100 ng/ml of shh, and 10 ng/mL of BMP4. The culture medium was replaced every day with PE induction media on days 7 to 11. To analyze the PE differentiation rate, the expression of EYA1 and HOXA3 was confirmed via immunofluorescence.

1. **Induction of** **Parathyroid-like Cells**

Parathyroid induction media is composed of SFBM supplemented with 50 ng/ml of FgF8b, 10 μM of SB431542, 0.1 μM of retinoic acid (RA), 100 ng/ml of shh, and 200 ng/ml of Noggin. On days 12 to 18, the medium was changed daily with parathyroid induction media. The differentiation rate of the parathyroid-like cells was measured at the end of differentiation. Details on the formulations and concentrations of the differentiation medium for each stage were provided in **Supplementary Table 1**.

1. **Effect of Extracellular Calcium on PTH Secretion**

ESCs derived parathyroid-like cells were exposed to normal calcium concentration medium (1.8 mM) or low calcium concentration medium (1 mM) for 7 days to check if cells were regulated by the extracellular calcium concentration. The detailed media formulation was shown in **Supplementary Table 1**. The cell supernatant was collected and the concentration of PTH was measured by ELISA after 0, 1, 3, 7 day of low/normal calcium culture.

1. **Lentivirus Transfection of hESCs**

The GCM2-overexpressing lentivirus and negative control lentivirus were purchased from GeneChem (Genechem, Shanghai, China). Briefly, undifferentiated H9 cells were dissociated with accutase (StemCell Technology, Canada), and then single cells were incubated with lentivirus (MOI＝5) and transfection reagent A/P in mTeSR medium supplemented with 10 μM Y27632. Transfection reagent A and P were commercial transfection reagents, which were also purchased from GeneChem company. They were improved substitutes for polybrene and used to assist lentivirus transfection. Puromycin was used to select stably transduced clones. Seven days after the screening, the transfection efficiency of lentivirus was evaluated by qPCR, western blot, and immunofluorescence.

1. **hESC-derived Parathyroid Organoids**

ESCs were differentiated into AFE cells in the 2D environment following our differentiation steps. Then the AFE cells were digested with trypsin-EDTA and washed with PBS. The Growth Factor Reduced (GFR) Basement Membrane Matrix (Corning) was thawed at 4 °C overnight. The AFE cells were then mixed gently with GFR matrigel at a density of 10 cells/µL on ice. 50ul of the matrigel/cells mixture was plated in the center of the pre-warmed 24-well culture plate. The matrigel was allowed to polymerize at 37 °C for 30 minutes and form 3D dome structures. 1 mL of organoid media was added per well to ensure the dome structures were all fully submerged. Organoid media was replaced every two days for a total of 21 days. The organoid medium was a mixture composed of SFBM supplemented with 100ng/ml of Noggin, 250ng/ml of R-Spondin-1, 3μM of CHIR99021, 50ng/ml of EGF, 50ng/ml of FgF8b, 10μM of SB431542, 0.1μM of RA, 100ng/ml of shh, and 10μM of Y27632.

1. **Patients and specimens**

Ethical approval was granted by Research Ethics Committee of the Tongji Medical University, and informed consent forms were signed by every patient. Three human parathyroid adenoma tissues and one human parathyroid carcinoma tissue were obtained from surgical resection samples at the Tongji Hospital of Huazhong University of Science and Technology. Fresh parathyroid adenoma tissues were fixed with paraformaldehyde, embedded in paraffin, and sectioned. For the primary culture of human parathyroid carcinoma cells, fresh tissue was minced and digested by collagenase and DNase solution, and single cell suspension was prepared using sterile 200 mesh sieves. Primary parathyroid carcinoma cells were seeded in the confocal dishes for the subsequent immunofluorescence experiments. Normal human parathyroid tissue sections were purchased from Shanghai Outdo Biotech Company. All human tissues, including human parathyroid adenoma, human parathyroid carcinoma and human parathyroid, were used as positive controls for immunofluorescence experiments.

1. **Immunofluorescence-paraffin embedded sections**

Human parathyroid adenoma and parathyroid paraffin embedded sections were placed in an oven at 65 °C for 2 hours. Antigen retrieval with EDTA buffer (PH 8.0) was done after deparaffinization and hydration. After washing, nonspecific binding was blocked with 10% donkey serum in PBS. The primary antibody was diluted in 5% donkey serum in PBS and incubated overnight at 4°C. The next day, secondary antibody was incubated after washing with PBS for three times. The antibodies were listed in the Supplementary Table 2. Nuclei were counterstained with DAPI, and the Tissue Autofluorescence Quenching Kit (Servicebio, G1221) was used to quench autofluorescence. After quenching tissue autofluorescence, the stained sections were photographed with a confocal microscope (OLYMPUS).

1. **Immunocytochemistry (ICC)**

Human ESCs were seeded into 24-well plates with cell-climbing slices (A thin glass sheet to which cells can adhere) at a density of 2 x 105 cells/cm2. At the end of each differentiation stage, cell climbing pieces were collected for immunofluorescence analysis of differentiation markers. Cells were washed with PBS buffer twice, then incubated with blocking solution (0.2% Triton and 10% donkey serum in PBS) for 50 minutes. Primary antibodies were diluted in 5% donkey serum in PBS and applied at 4°C overnight. The fluorescein-conjugated secondary antibodies were incubated for one hour at room temperature. All antibodies used were listed in the Supplementary Table 2. DAPI was used for nucleus staining. Confocal images were obtained with the Olympus Confocal FluoView1000 microscope. Cell counts were performed in ImageJ, and the percentage of positive cell (cell differentiation rate) = Number of positive cells/Number of DAPI positive cells.

1. **Immunofluorescence-Frozen organoid sections**

Organoid were washed with PBS twice and then fixed with 4% paraformaldehyde for 30 minutes at room temperature. After washing three times with PBS, organoids were carefully collected with a cell scraper into a 20% sucrose solution. It took approximately 2 days for organoids to sink to the bottom at 4°C. Parathyroid organoids were first embedded in 3% agarose and then snap-frozen in optimal cutting temperature (OCT) compound. Frozen organoid blocks were stored at −80 °C and sectioned with a thickness of 10 μm. Frozen sections were washed with PBS first and then blocked with 5% donkey serum in PBS solution containing 0.2% Triton X-100. The next steps were similar to the ICC experiment protocols. Photographs were taken and analyzed by OLYMPUS confocal microscopy. The quantitative method of positive cell percentage was the same as for the ICC experiments.

1. **Quantitative real-time PCR**

The total RNA was extracted by using RNA extraction kits (Accurate Biotechnology). Following quantification, cDNA was synthesized with the Reverse Transcription kit (Accurate Biotechnology). The real-time quantitative PCR amplifications were performed by using the CFX96 PCR System (Bio-Rad). The primer sequences were derived from PrimerBank Database and purchased from the Tsingke company. All PCR primer sequences were listed in the Supplementary Table 3. The relative mRNA expression was calculated by using the 2 −ΔΔCt method normalized to GAPDH.

1. **Western Blots**

Proteins were extracted from the human ESCs by using RIPA buffer and quantified by the Coomassie Brilliant Blue method. Then the total proteins were electrophoresed on SDS-PAGE gels before being transferred to polyvinylidene fluoride (PVDF) membranes. The PVDF membranes were incubated with primary antibodies diluted with 0.5% BSA/TBST overnight at 4 °C. Secondary antibodies were anti-rabbit IgG-HRP and anti-mouse IgG-HRP. The WesternBright ECL kit (Advansta) was used for visualization.

1. **ELISA**

Parathyroid hormone intact ELISA kit (NM59041) was purchased from IBL (Germany). PTH was detected using the double antibody sandwich method. In brief, if PTH were present in the cell culture supernatant, it would bind to the antibodies to form an antigen-antibody complex like this: streptavidin well, biotinylated PTH (39-84) antibody, intact PTH, and HRP conjugated PTH (1-34) antibody. Absorbance data were acquired with a microplate reader at the wavelength of 450nm. By multiplying the PTH concentration with the liquid volume (2ml) of one well of the six-well plate, the total PTH can be obtained from the cell culture supernatant. By multiplying the total number of cells in one well of the six-well plate with the percentage of PTH positive cells, the number of hESC-PT cells can be obtained. The daily PTH secretion per hESC-PT cell (fg/cell*day) can be obtained by dividing the total PTH by the number of hESC-PT cells and then the number of days.

1. **RNA Bulk Sequencing and RNA Sequencing Analysis**

Human ESC cells (H9) were differentiated into hESC-PT by following our proposed differentiation protocol. Human ESC (n=5) and human ESC-derived PT cells (n=5) were lysed and total RNA was extracted. RNA quality (RIN) was determined using High Sensitivity RNA Screen-Tapes and all 10 RNA samples were suitable for library preparation. These libraries were sequenced using the Illumina NovaSeq 6000 platform. For the differential gene expression analysis, the expression levels of parathyroid differentiation-related genes and stemness related genes were first taken Log10, and then the expression differences of the stem cells before and after differentiation were calculated. A heatmap was generated with GraphPad Prism.

1. **Statistical analysis**

Statistical analyses were performed by GraphPad Prism (GraphPad Software Inc.). All experiments were repeated for at least 3 times independently. The differentiation percentage of positive cells was calculated by dividing the number of positive cells by the total cell number. More than three visual fields were selected randomly in immunofluorescence experiments, and the cells were counted by using ImageJ. Paired/unpaired T tests were performed for comparing means between two matched/unmatched groups, and one-way ANOVA was used for making comparisons among three or more groups. Multiple parameters between two or more groups were compared using the two-way ANOVA. P < 0.05 was considered as statistically significant.
