# Supplementary figures and images for: Directed differentiation of human embryonic stem cells into parathyroid cells and establishment of parathyroid organoids
Source: Cell Prolif. 2024 Mar 18;57(8):e13634. doi: 10.1111/cpr.13634 (PMC11294423; doi:10.1111/cpr.13634)

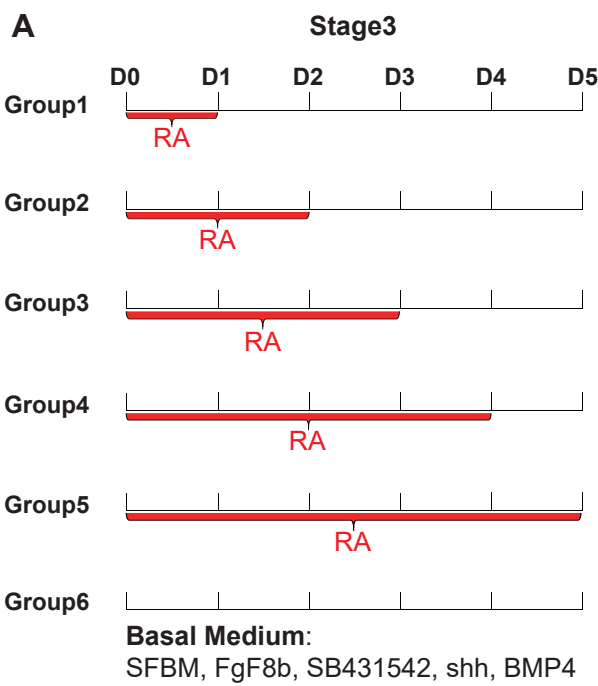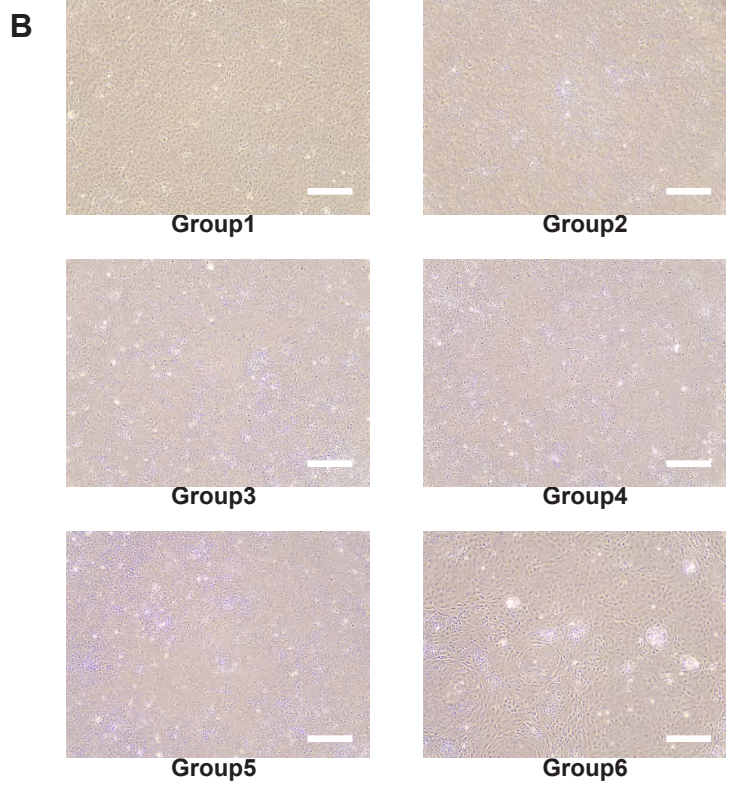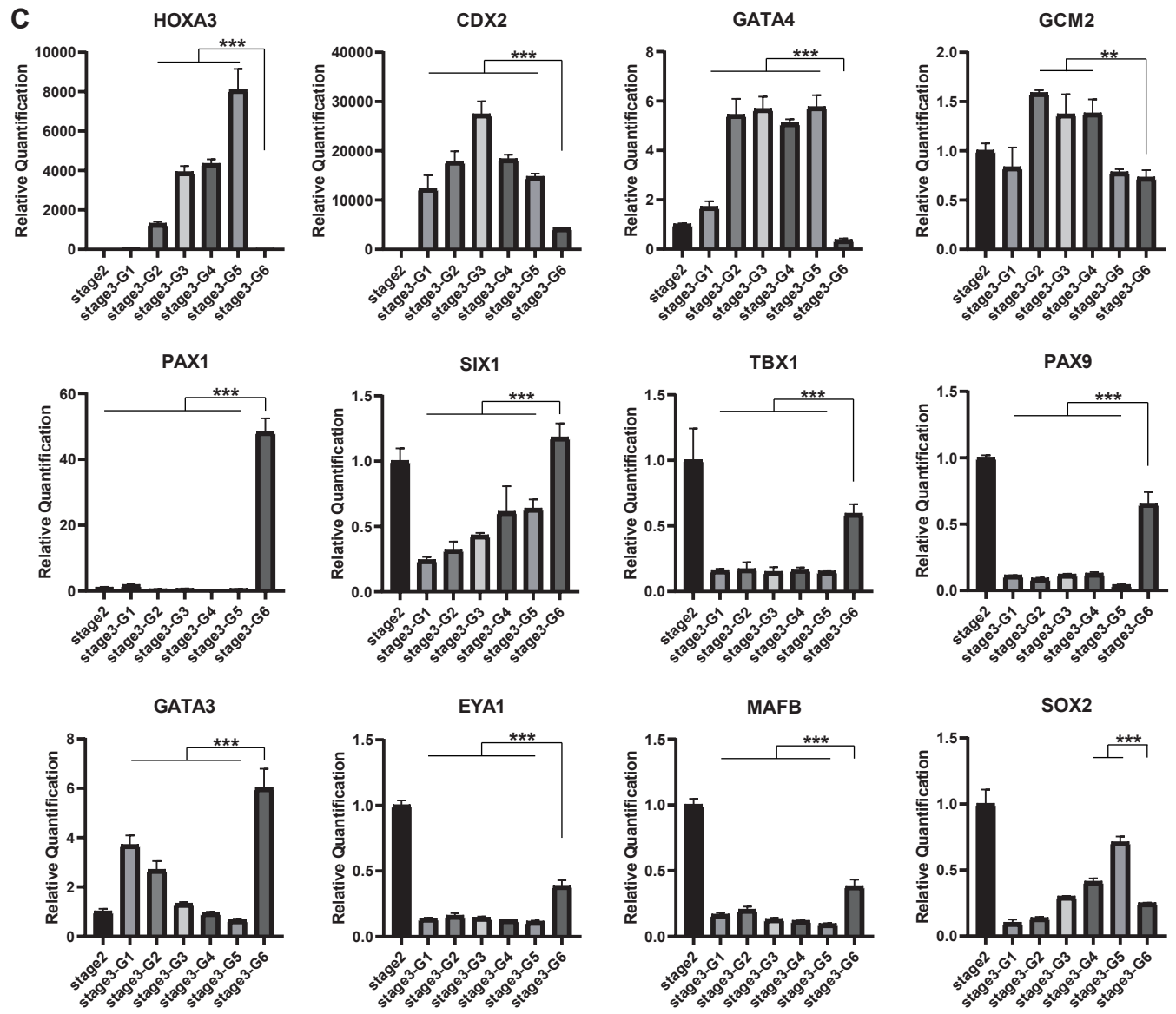

Supplement: Supplementary file 2 — Figure S1. The effects of different durations of RA exposure on pharyngeal endoderm differentiation. (A) Schematic diagram of different RA action time in each group. (B) Typical white light pictures of each group at the end of pharyngeal endoderm differentiation. (C) RT‐qPCR showed different expression of HOXA3, CDX2, GATA4, GCM2, PAX1, SIX1, TBX1, PAX9, GATA3, EYA1, MAFB and SOX2 in the different durations of RA exposure groups. RA, retinoic acid. Scale bar: 200 μm (B). Statistics: Data are presented as means ± SEM. (C) one‐way ANOVA with Tukey's multiple comparisons. **p < 0.01, ***p < 0.001. [file CPR-57-e13634-s009.pdf]

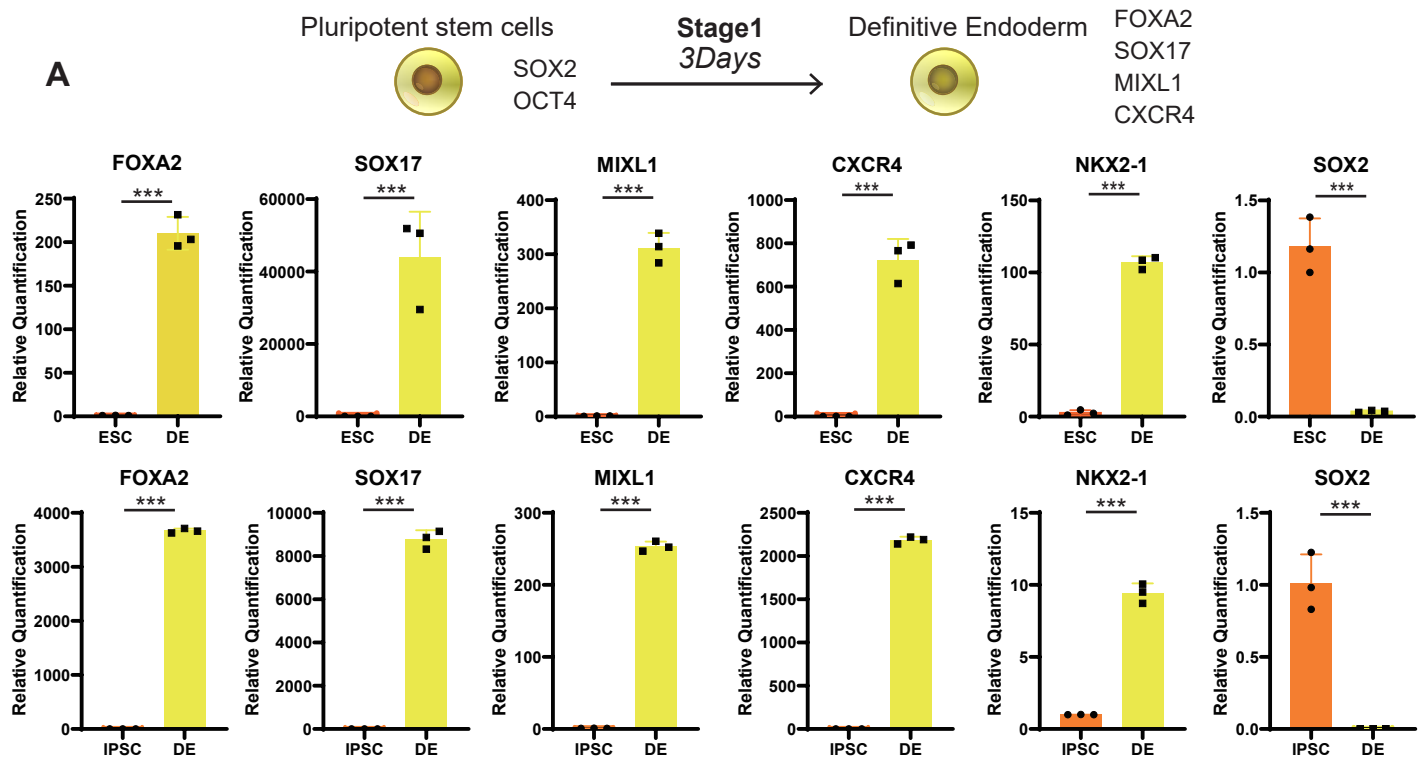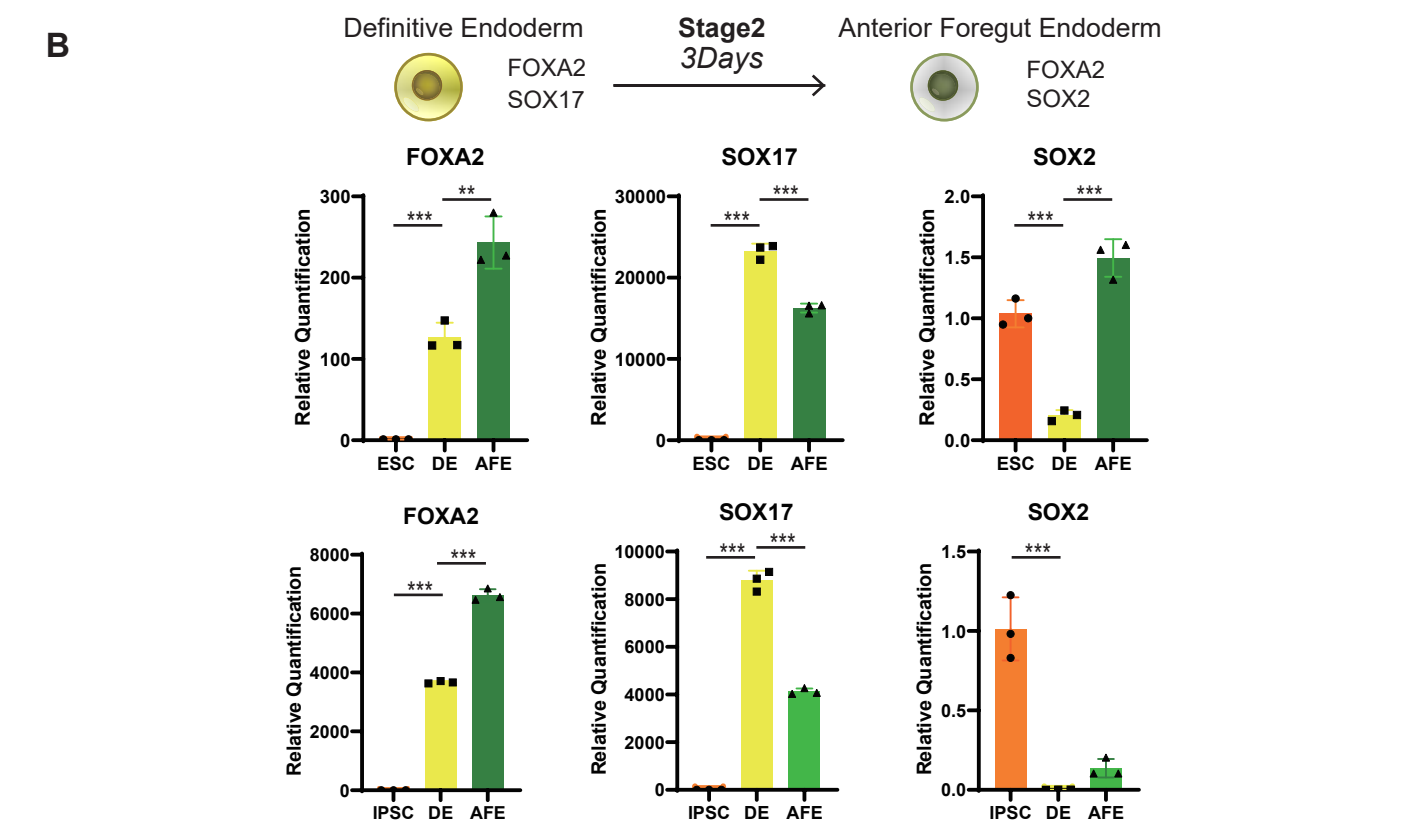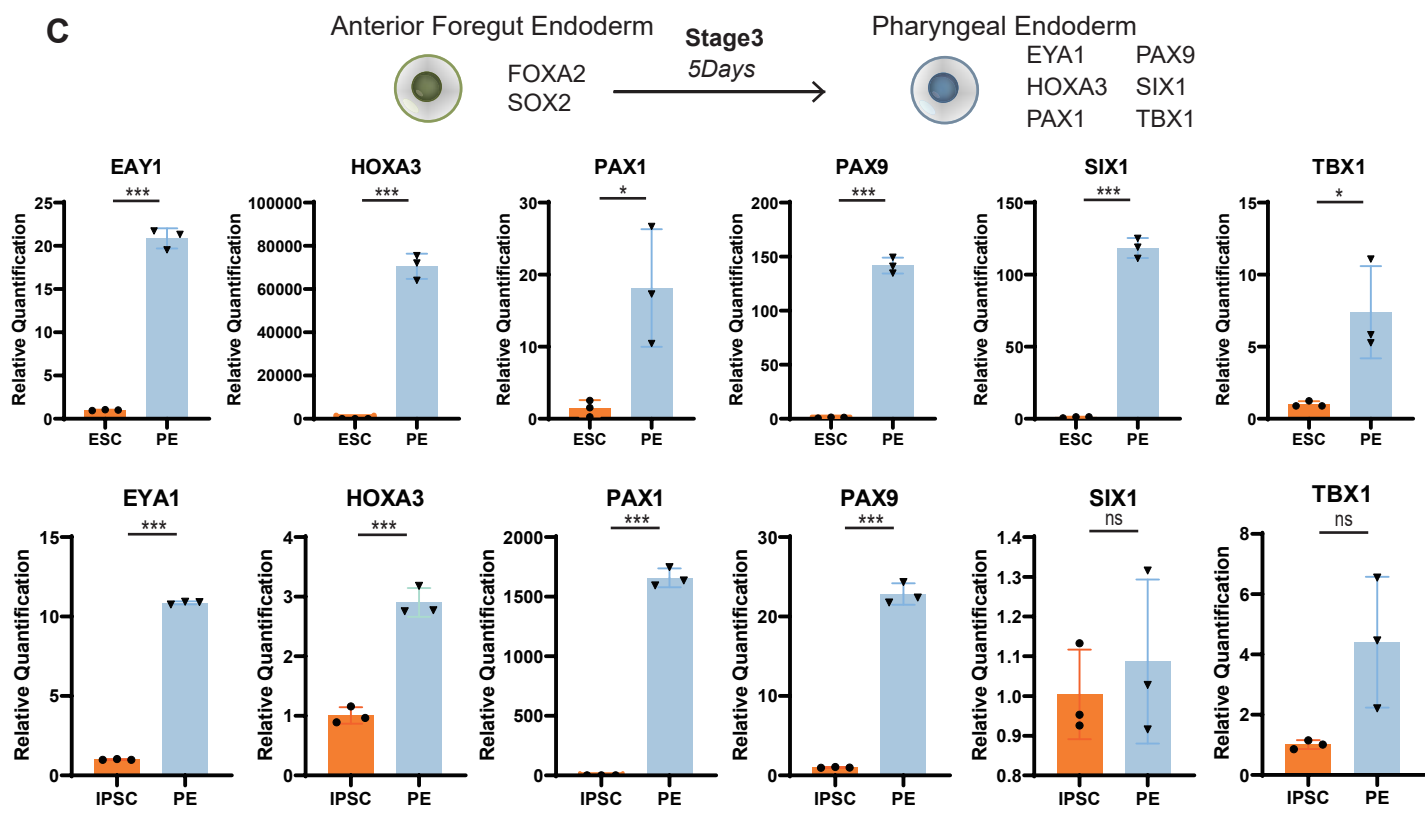

Supplement: Supplementary file 3 — Figure S2. Successful expression of the stage I/II/III differentiation markers. (A) RT‐qPCR showed expression of DE‐related transcription factor genes FOXA2, SOX17, MIXL1, CXCR4, NKX2‐1 as well as hESC‐associated genes SOX2. (B) RT‐qPCR showed different expression of FOXA2, SOX17 and SOX2 in ESC/IPSC, DE and AFE stages. (C) RT‐qPCR showing expression of PE‐related transcription factor genes EYA1, HOXA3, PAX1, PAX9, SIX1 and Tbx1. ESC, embryonic stem cell; IPSC, induced pluripotent stem cell; DE, definitive endoderm; AFE, anterior foregut endoderm; PE, pharyngeal endoderm. Statistics: Data are presented as means ± SEM. (A,C) Paired two‐sided t‐test; (B) one‐way ANOVA with Tukey's multiple comparisons. *p < 0.05, **p < 0.01, ***p < 0.001. [file CPR-57-e13634-s015.pdf]

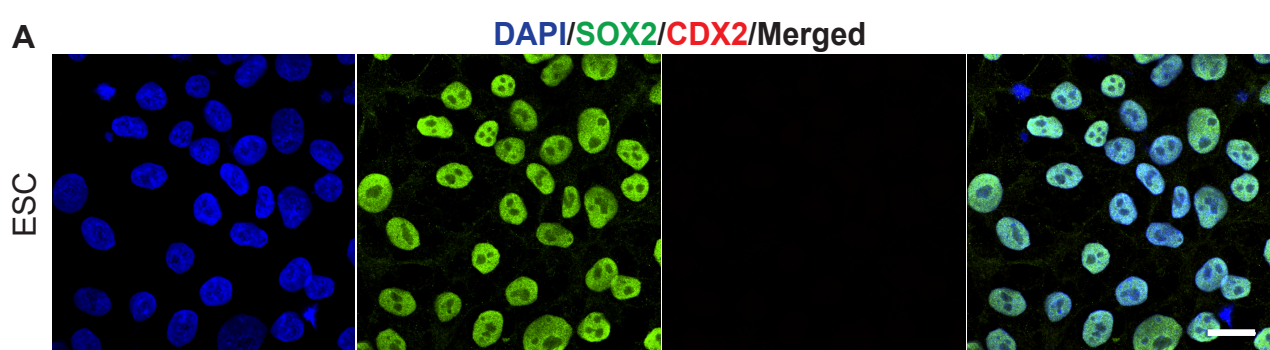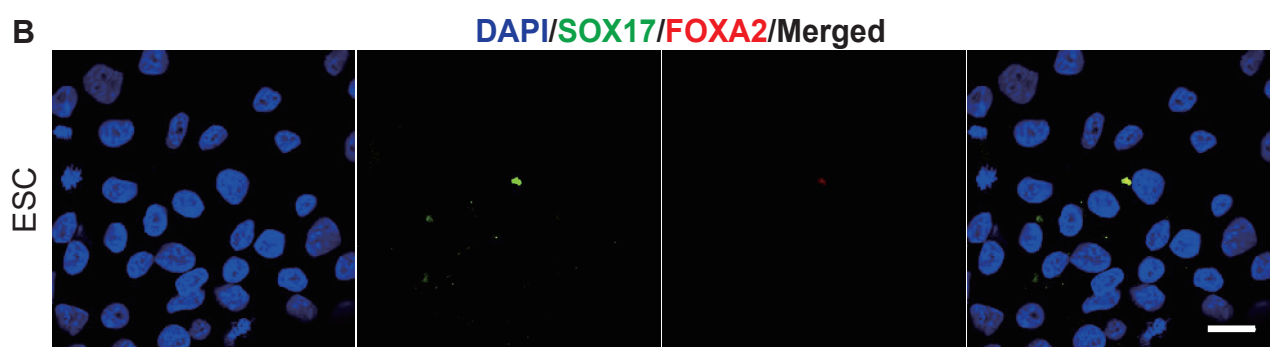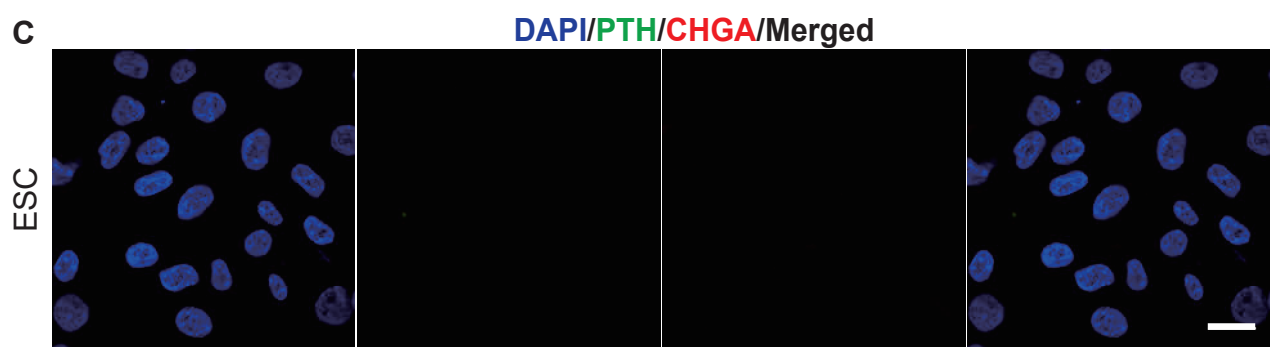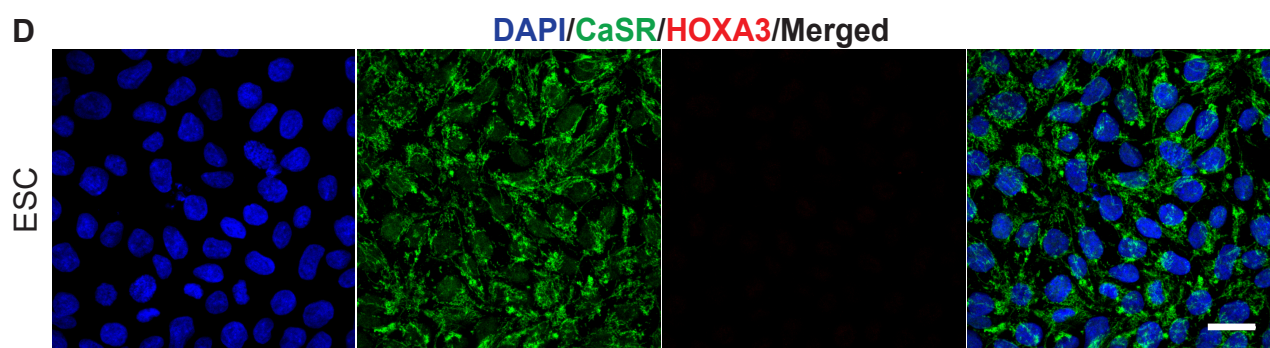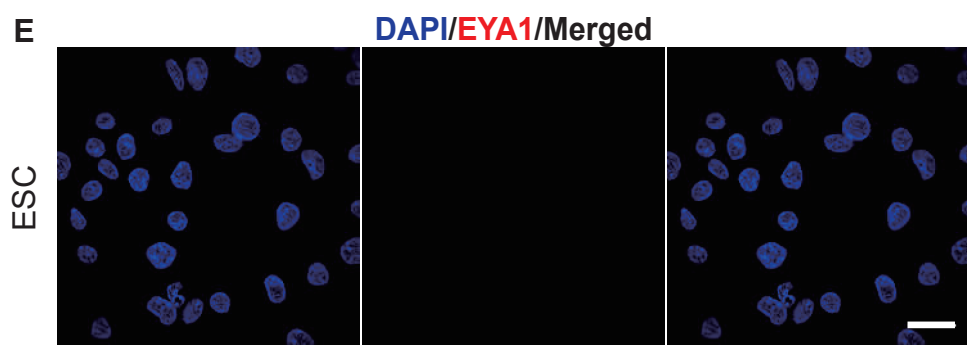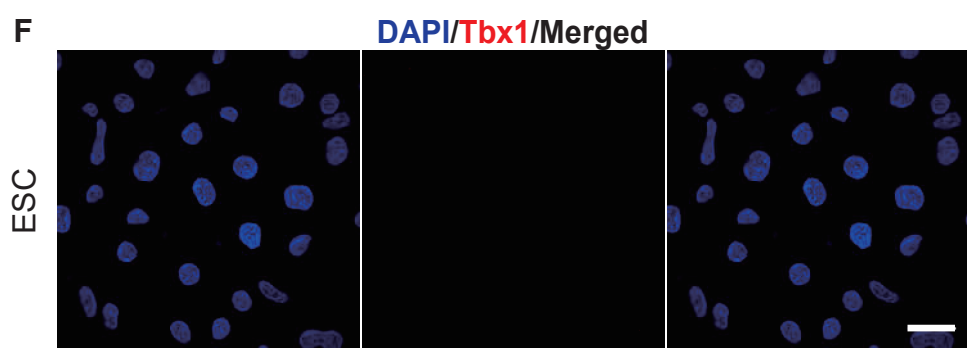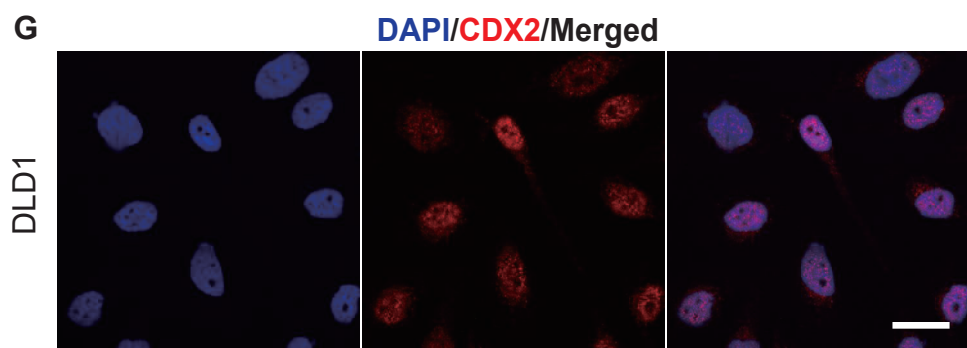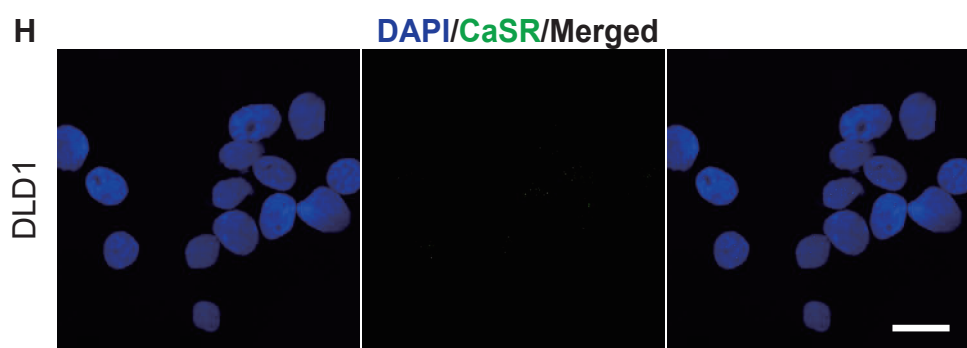

Supplement: Supplementary file 4 — Figure S3. Expression of differentiation markers were measured with immunofluorescence as negative/positive controls. (A) Co‐immunostaining of SOX2 with CDX2 in ESCs. (B) Co‐immunostaining of SOX17 with FOXA2 in ESCs. (C) Co‐immunostaining of PTH with CHGA in ESCs. (D) Co‐immunostaining of CaSR with HOXA3 in ESCs. (E) Immunostaining of EYA1 in ESCs. (F) Immunostaining of Tbx1 in ESCs. (G) Immunostaining of CDX2 in DLD1 cells. (H) Immunostaining of CaSR in DLD1 cells. ESCs, embryonic stem cells. Scale bar: 20 μm (A–F); 30 μm (G,H). [file CPR-57-e13634-s012.pdf]

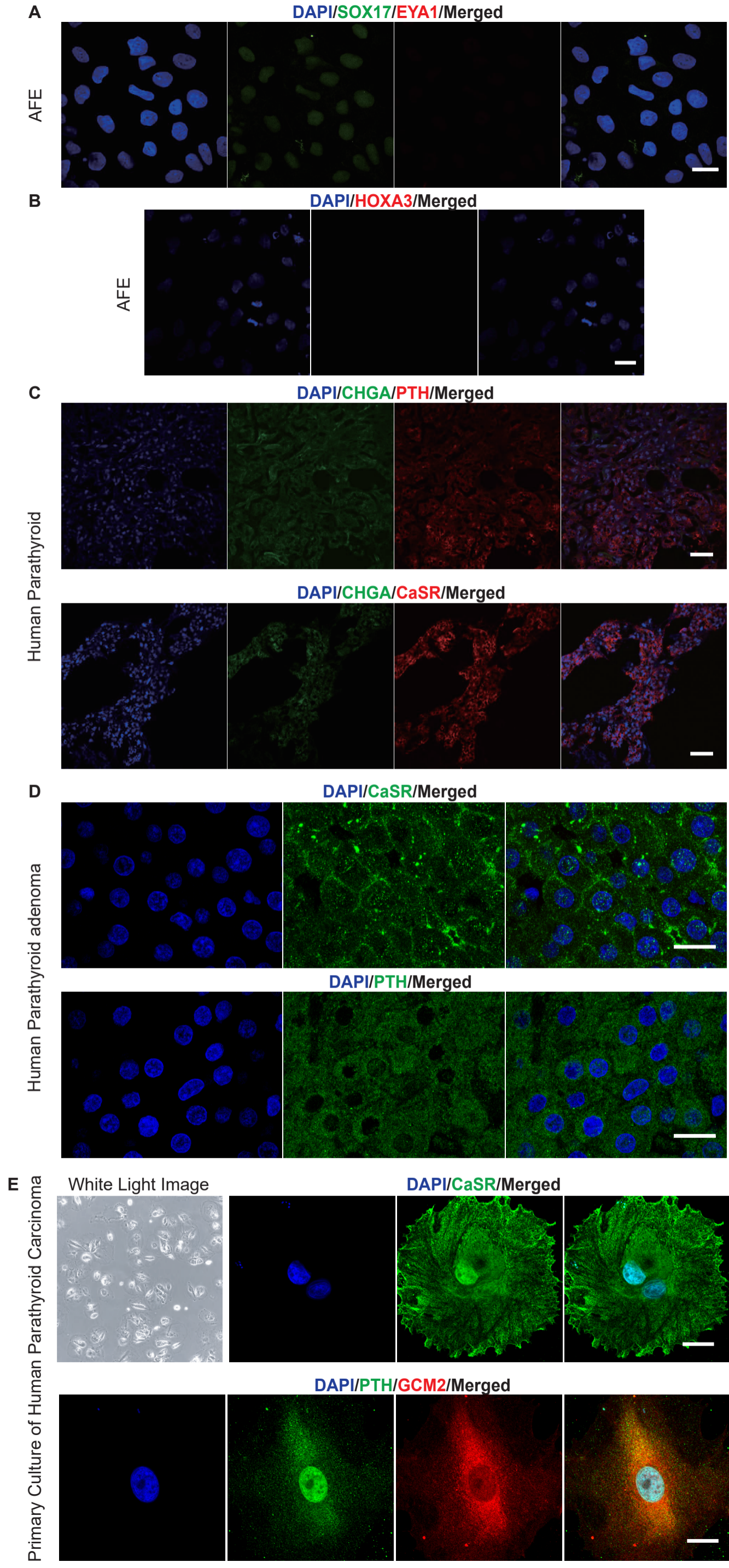

Supplement: Supplementary file 5 — Figure S4. For immunofluorescence studies, AFE was used as the negative control while human parathyroid, parathyroid adenoma and parathyroid carcinoma were used as positive controls. (A) Co‐immunostaining of SOX17 with EYA1 in AFE. (B) Immunostaining of HOXA3 in AFE. (C) Co‐immunostaining of PTH, CaSR with CHGA in human parathyroid. (D) Immunostaining of PTH and CaSR in human parathyroid adenoma. (E) The white light image and immunofluorescence staining of primary parathyroid carcinoma cells. AFE, anterior foregut endoderm. Scale bar: 100 μm (C); 20 μm (A, B, E); 10 μm (D). [file CPR-57-e13634-s004.pdf]

**A**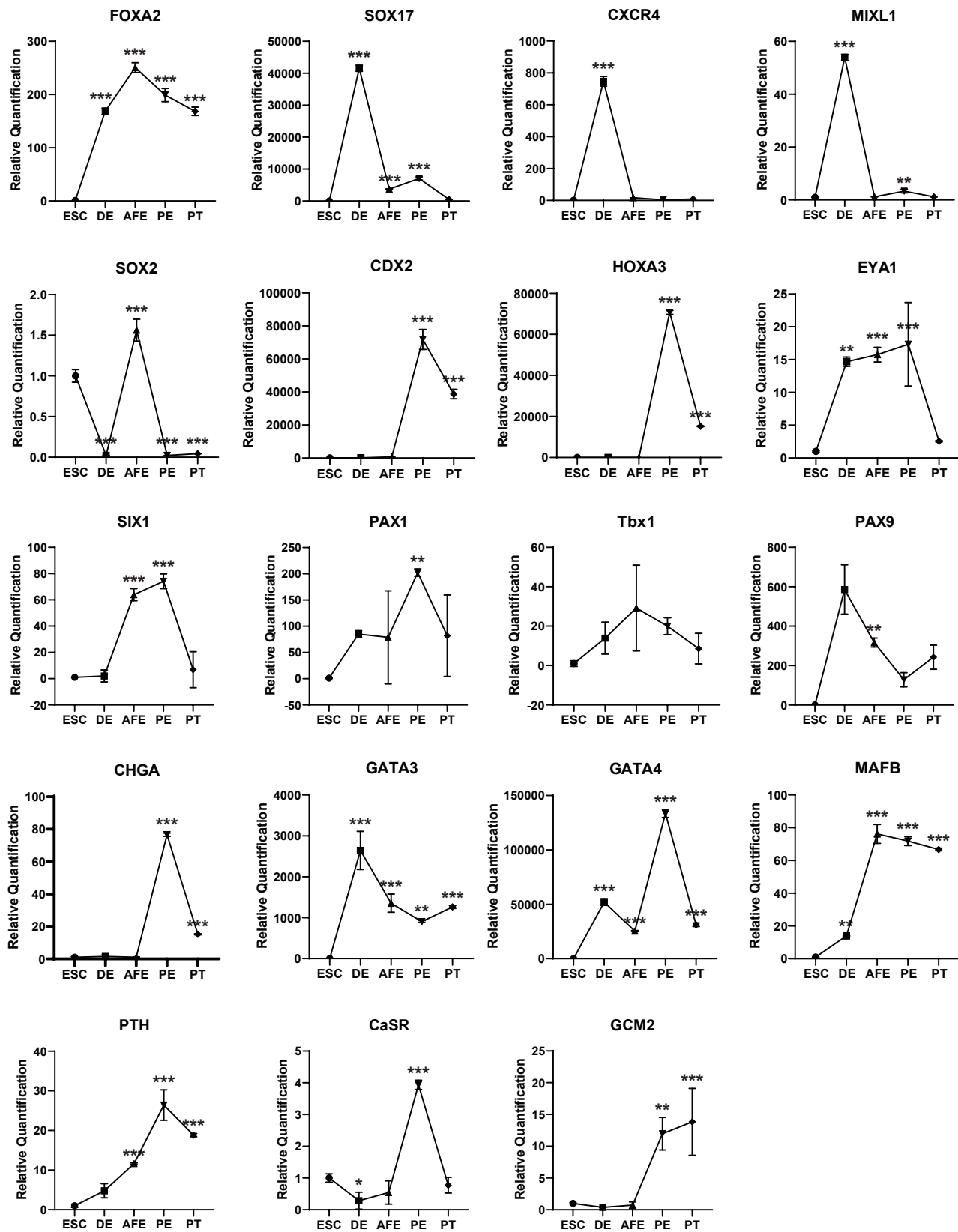

Supplement: Supplementary file 6 — Figure S5. Time course of the differentiation markers at different stages of parathyroid differentiation in vitro. (A) RT‐qPCR showed the time course of differentiation markers in ESC, DE, AFE, PE and PT stages. ESC, embryonic stem cell; DE, definitive endoderm; AFE, anterior foregut endoderm; PE, pharyngeal endoderm; PT, human embryonic stem cells‐derived parathyroid‐like cells. Statistics: Data are presented as means ± SEM. (A) one‐way ANOVA with Tukey's multiple comparisons. *p < 0.05, **p < 0.01, ***p < 0.001. [file CPR-57-e13634-s007.pdf]

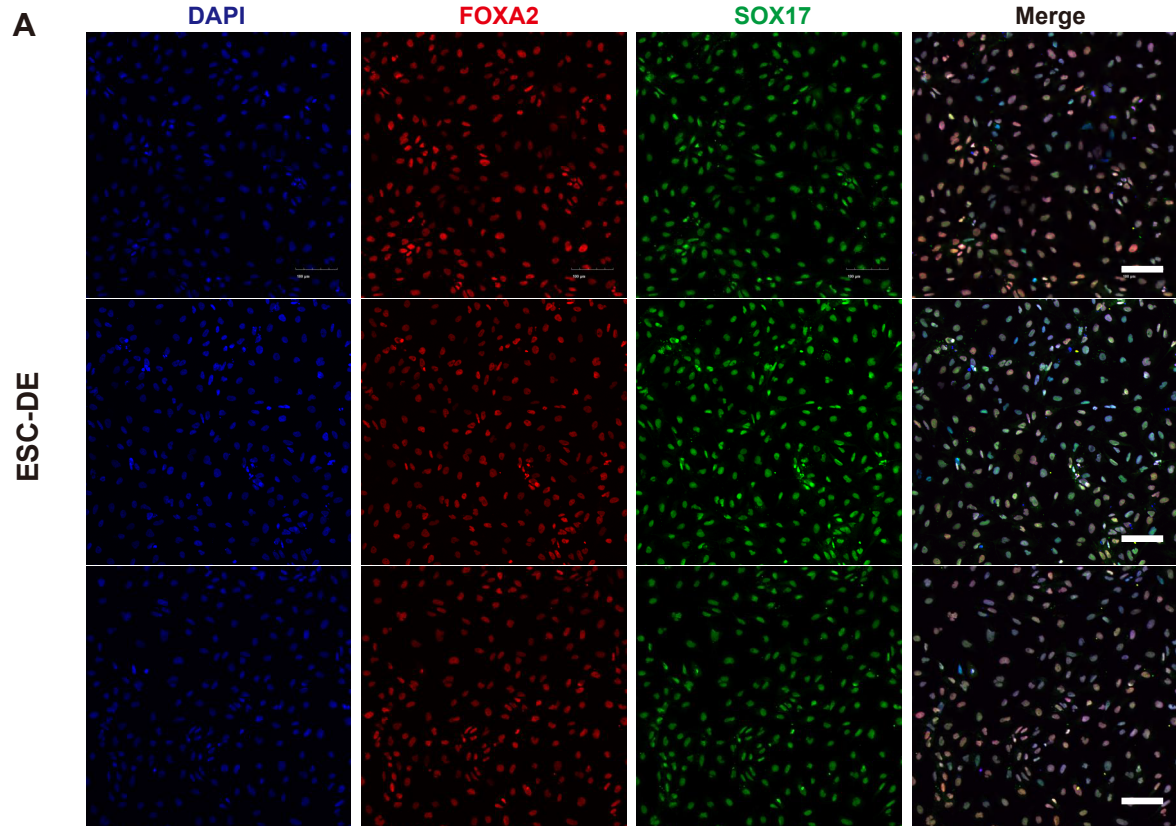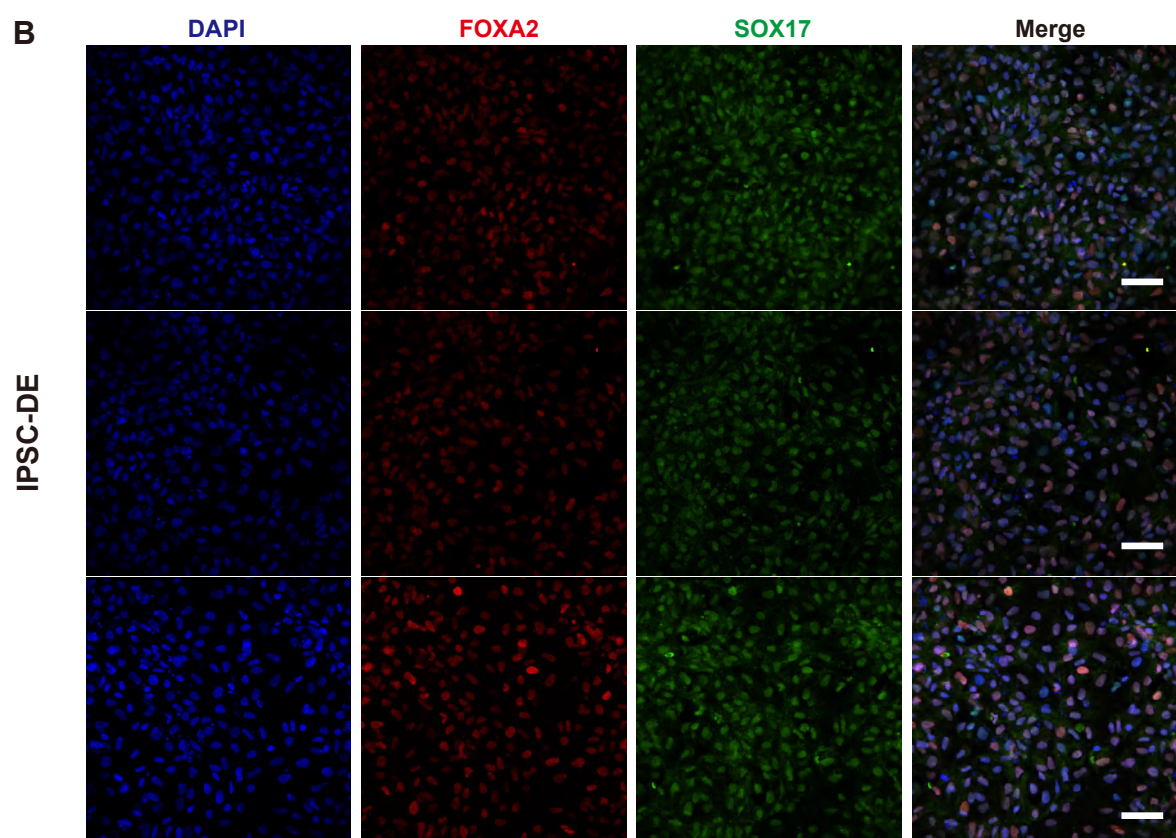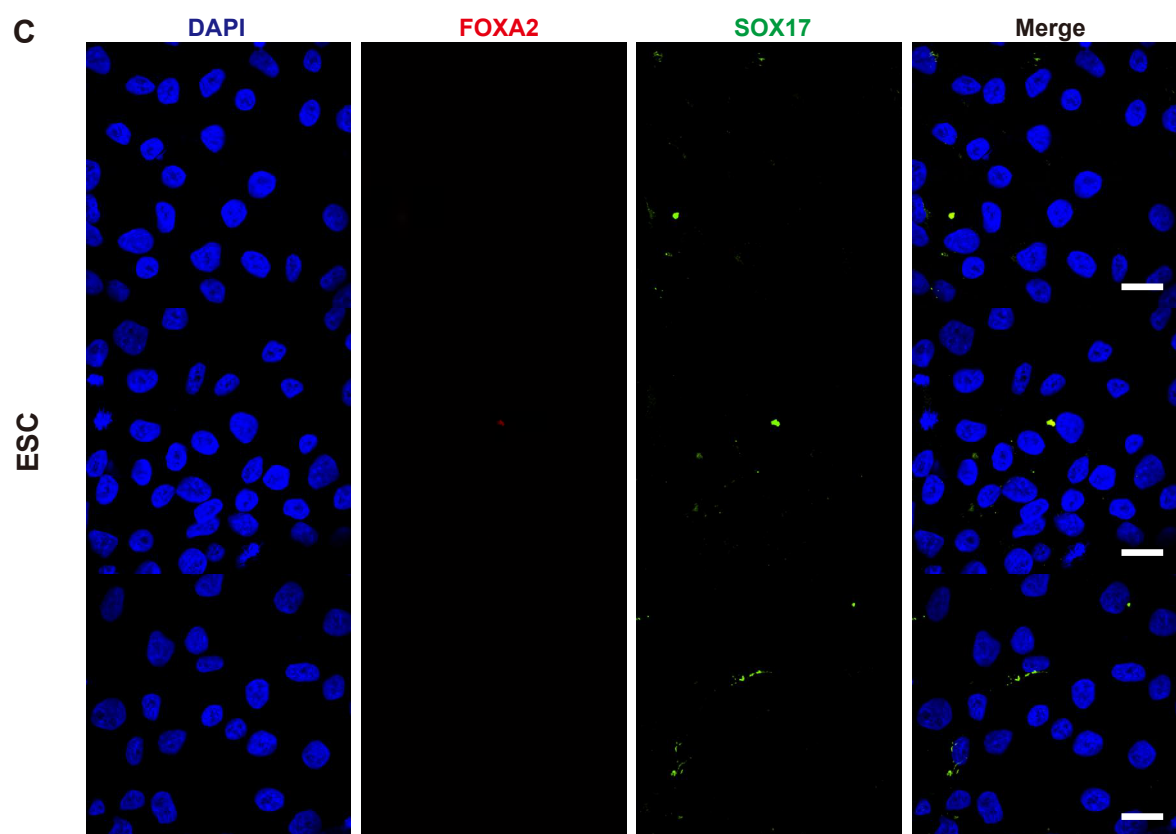

Supplement: Supplementary file 7 — Figure S6. Selection of immunofluorescence visual field and quantification of the percentage of FOXA2‐SOX17 double‐positive cells in the DE stage. (A) Co‐immunostaining of FOXA2 with SOX17 in ESC‐derived DE cells. (B) Co‐immunostaining of FOXA2 with SOX17 in IPSC‐derived DE cells. (C) Co‐immunostaining of FOXA2 with SOX17 in ESC. Scale bar: 100 μm (A); 50 μm (B); 20 μm (C). [file CPR-57-e13634-s010.pdf]

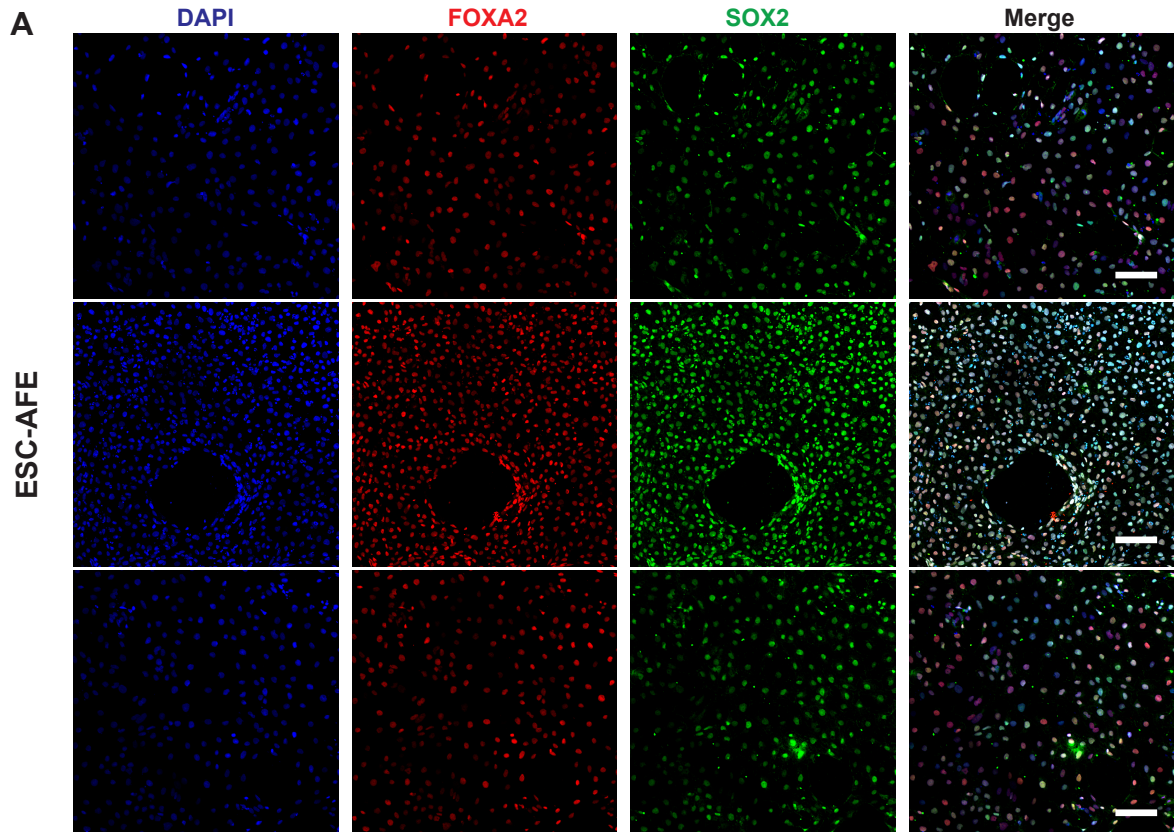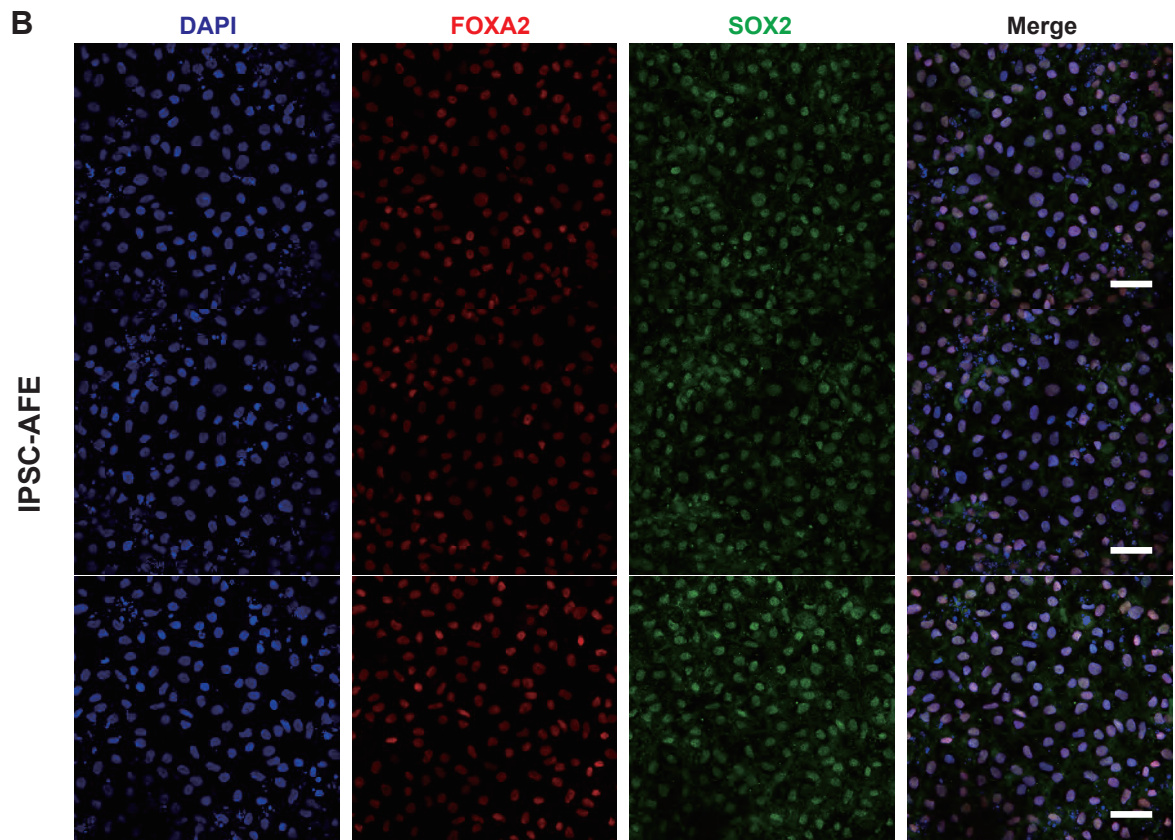

Supplement: Supplementary file 8 — Figure S7. Selection of immunofluorescence visual field and quantification of the percentage of FOXA2‐SOX2 double‐positive cells in the AFE stage. (A) Co‐immunostaining of FOXA2 with SOX2 in ESC‐derived AFE cells. (B) Co‐immunostaining of FOXA2 with SOX2 in IPSC‐derived AFE cells. Scale bar: 100 μm (A); 50 μm (B). [file CPR-57-e13634-s014.pdf]

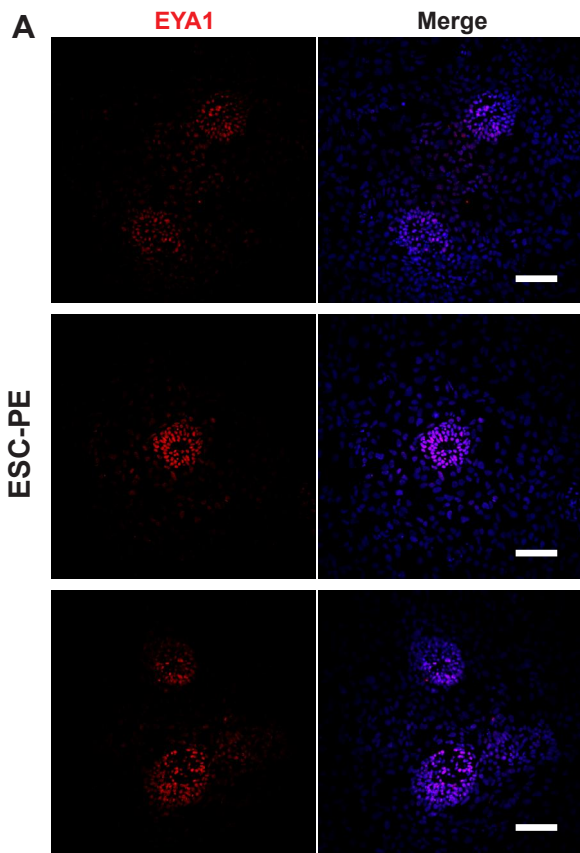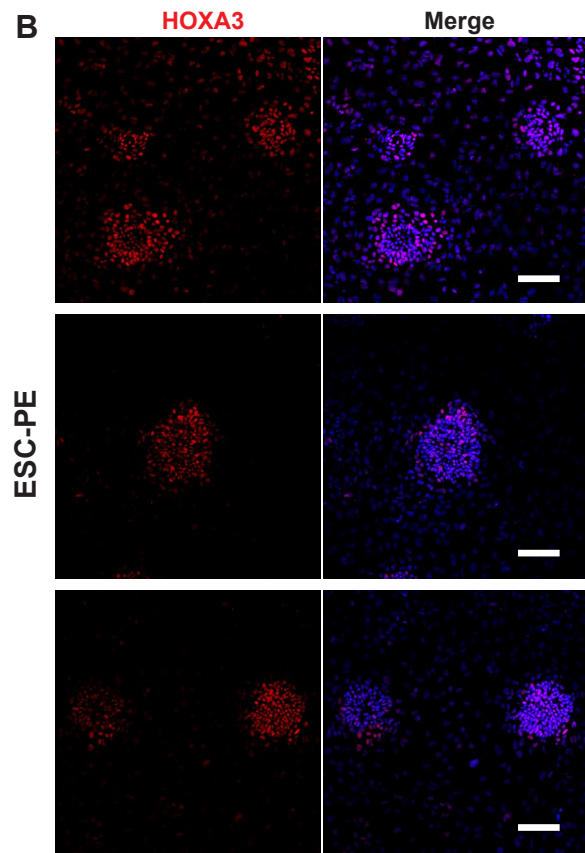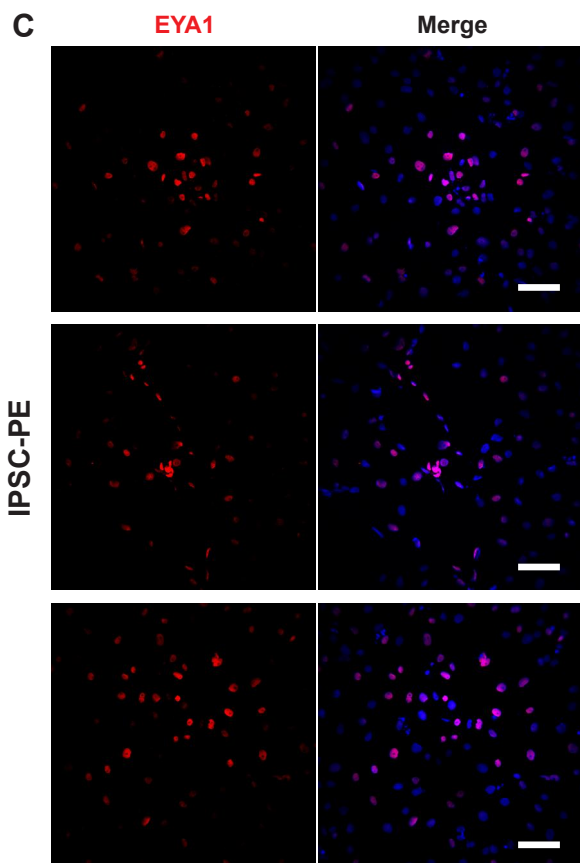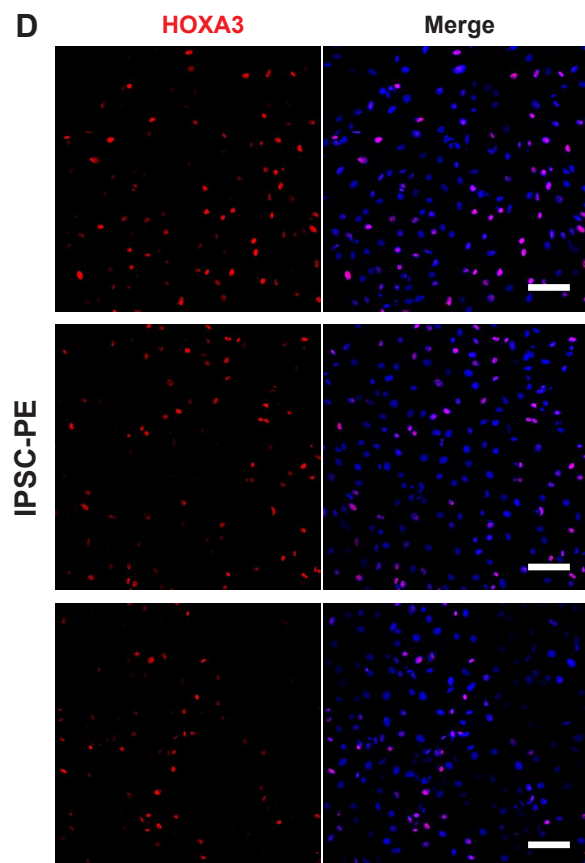

Supplement: Supplementary file 9 — Figure S8. Selection of immunofluorescence visual field and quantification of the percentage of EYA1/HOXA3 positive cells in the PE stage. (A) Immunostaining of EYA1 in ESC‐derived PE cells. (B) Immunostaining of HOXA3 in ESC‐derived PE cells. (C) Immunostaining of EYA1 in IPSC‐derived PE cells. (D) Immunostaining of HOXA3 in IPSC‐derived PE cells. Scale bar: 100 μm (A–D). [file CPR-57-e13634-s006.pdf]

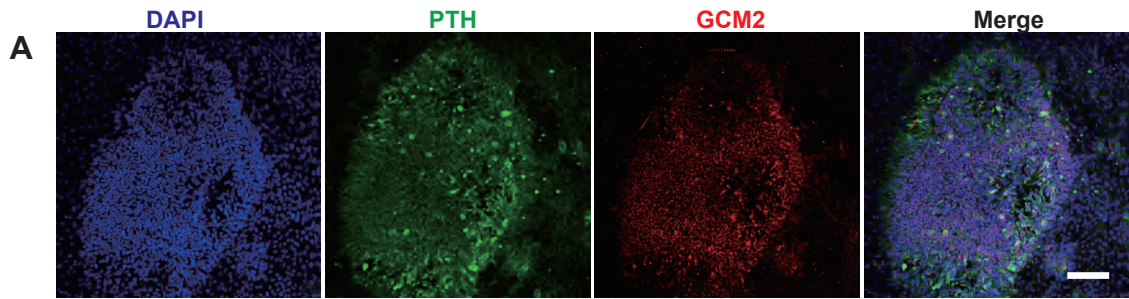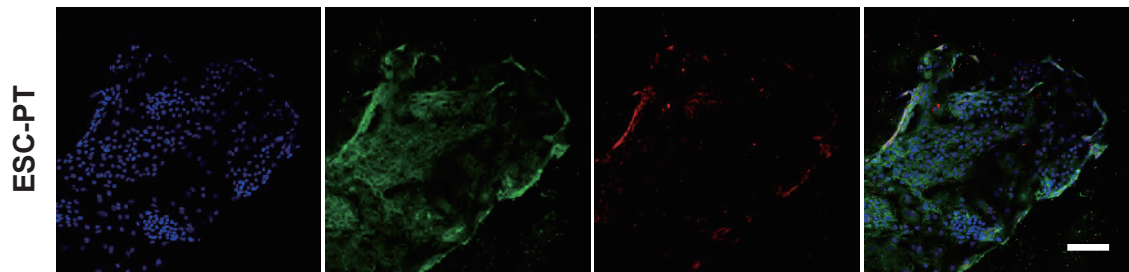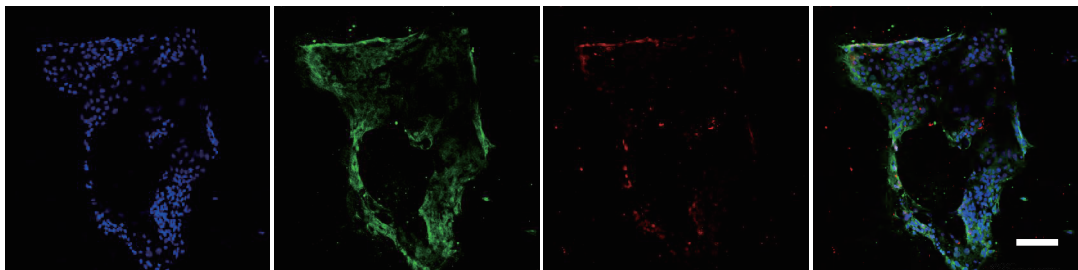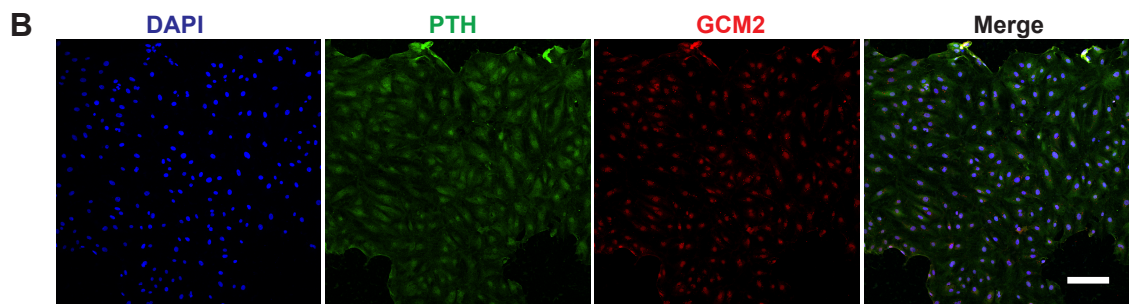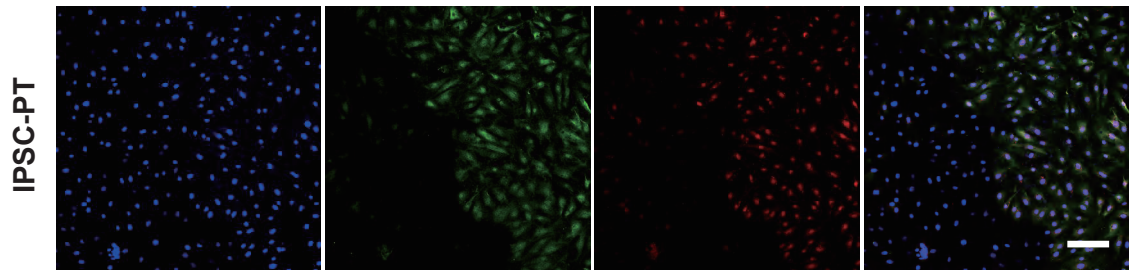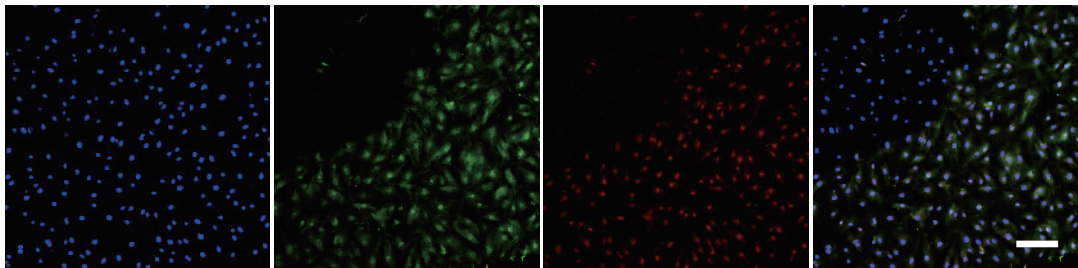

Supplement: Supplementary file 10 — Figure S9. Selection of immunofluorescence visual field and quantification of the percentage of PTH‐GCM2 double positive cells in the PT stage. (A) Co‐immunostaining of PTH with GCM2 in ESC‐derived PT cells. (B) Co‐immunostaining of PTH with GCM2 in IPSC‐derived PT cells. Scale bar: 100 μm (A); 200 μm (B). [file CPR-57-e13634-s002.pdf]

**A**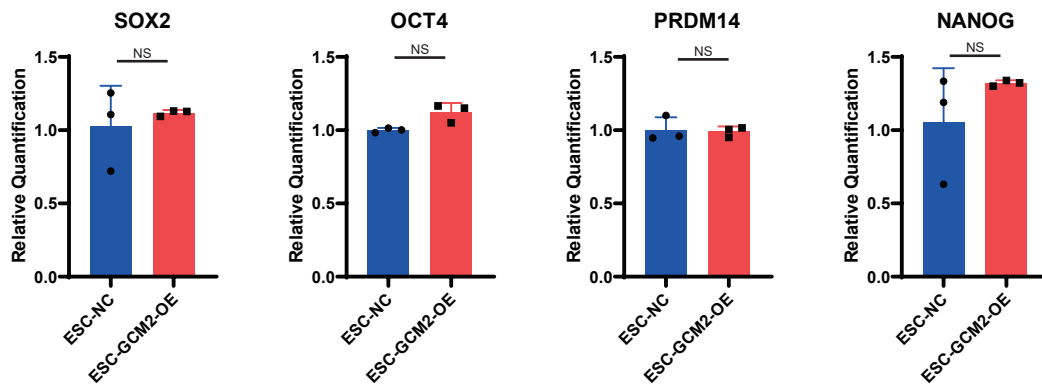**B**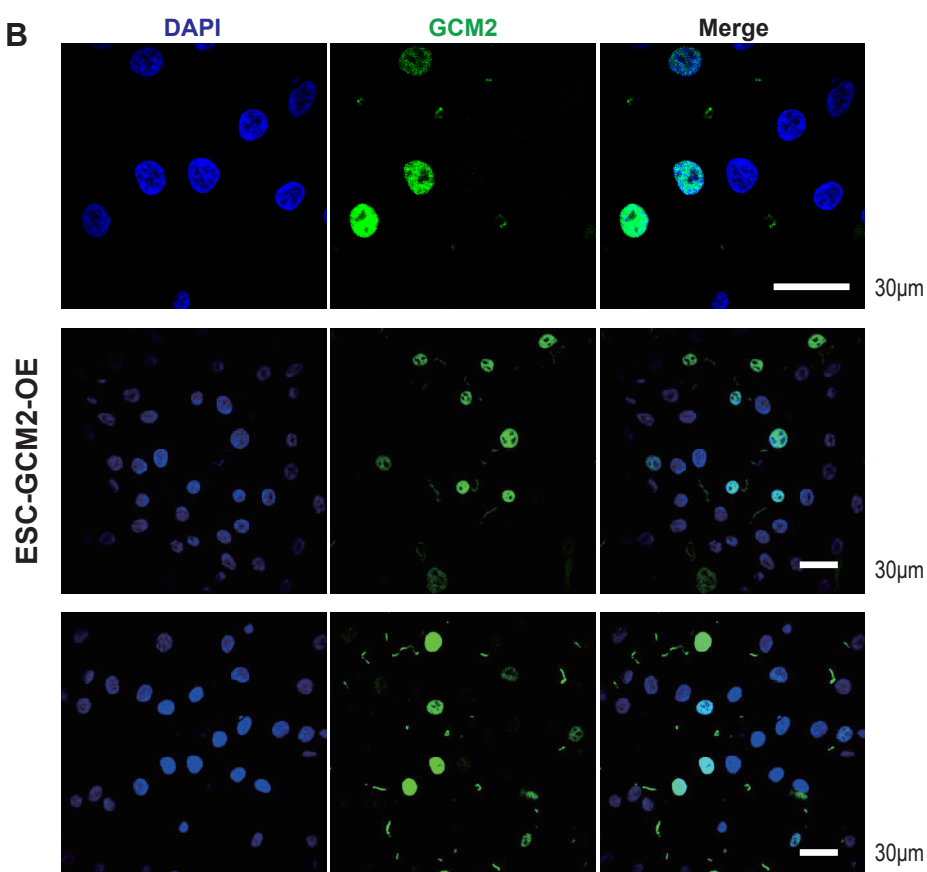**C**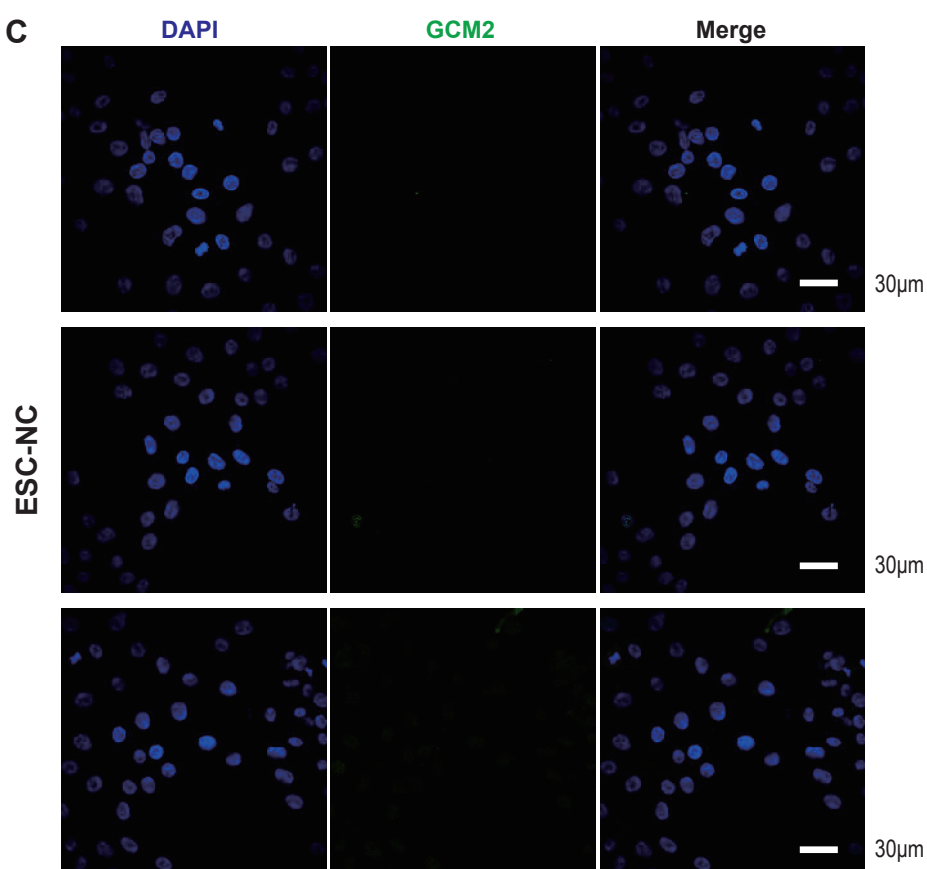**D**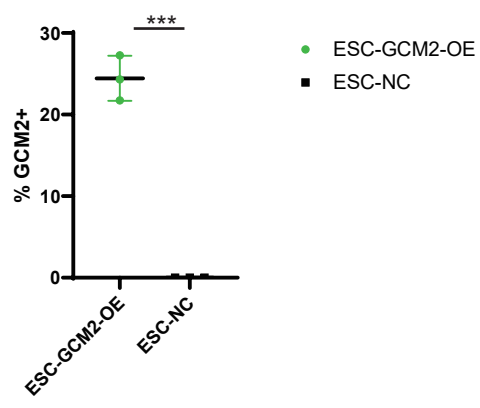

Supplement: Supplementary file 11 — Figure S10. Selection of immunofluorescence visual field and quantification of the lentiviral transfection efficiency. (A) RT‐qPCR showed relative quantification of NANOG, OCT4, PRDM14 and SOX2 in ESC‐NC and ESC‐GCM2‐OE cells. (B) Immunostaining of GCM2 in ESC transfected with GCM2‐overexpression lentivirus. (C) Immunostaining of GCM2 in ESC transfected with negative control lentivirus. (D) Quantification of the lentiviral transfection efficiency. Scale bar: 30 μm (A,B). Statistics: Data are presented as means ± SD. (A,D) unpaired two‐sided t‐test. ***p < 0.001 and NS, not significant. [file CPR-57-e13634-s003.pdf]

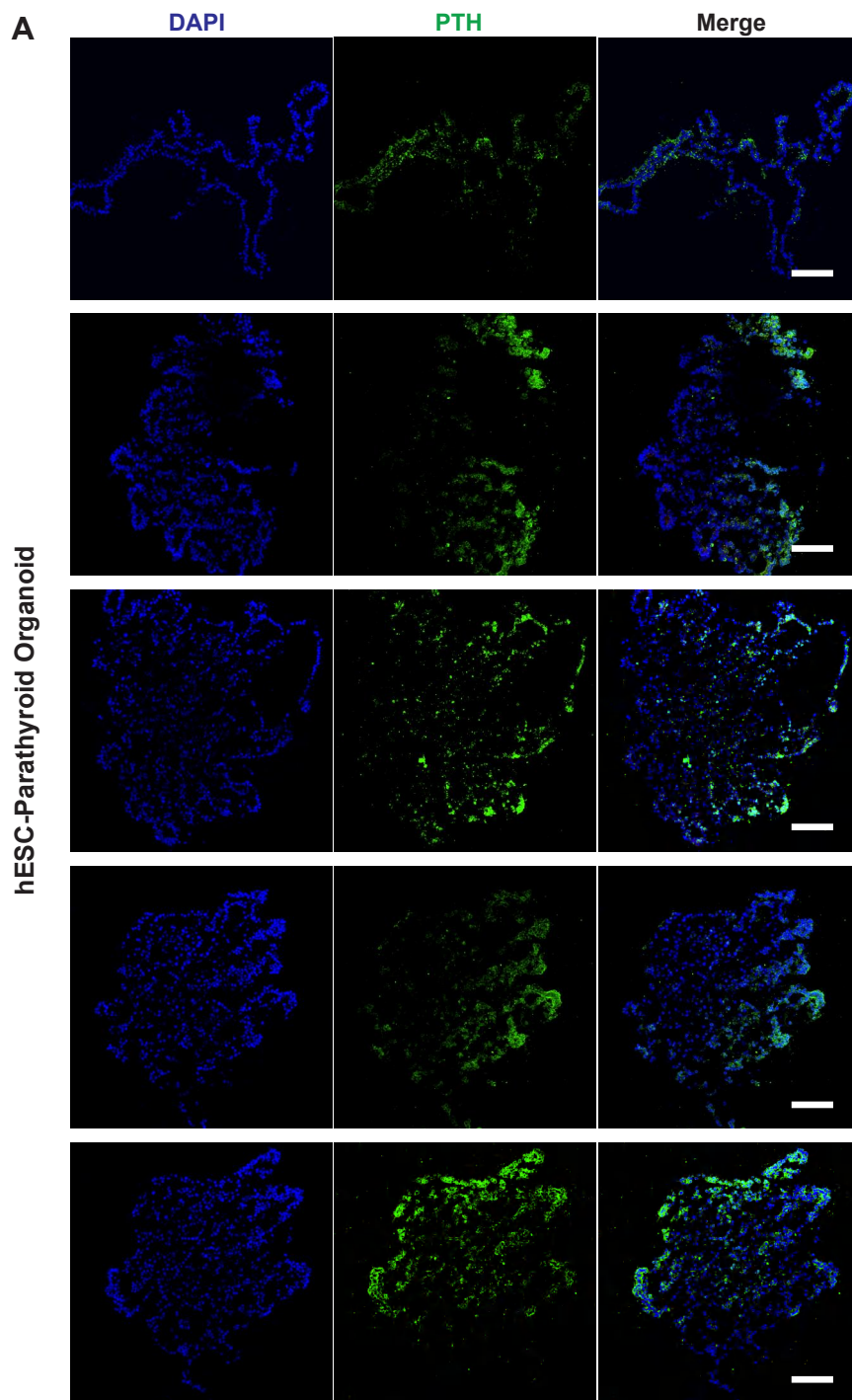

Supplement: Supplementary file 12 — Figure S11. Selection of immunofluorescence visual field and quantification of the percentage of PTH‐positive cells in the hESC‐derived parathyroid organoids. (A) Immunostaining of PTH in ESC‐derived parathyroid organoids. Scale bar: 100 μm (A). [file CPR-57-e13634-s011.pdf]
