# Supplementary material for: Directed differentiation of human embryonic stem cells into parathyroid cells and establishment of parathyroid organoids
Source: Cell Prolif. 2024 Mar 18;57(8):e13634. doi: 10.1111/cpr.13634 (PMC11294423; doi:10.1111/cpr.13634)
Supplement: Supplementary file 13 — Table S1. Media formulations for stem cell differentiation and organoid culture. Table S1A. The formulation of 500 mL serum‐free basal medium (SFBM). Table S1B. The formulation of definitive endoderm induction media (Day 1 to Day 3). Table S1C. The formulation of anterior foregut endoderm induction media (Day 4 to Day 6). Table S1D. The formulation of pharyngeal endoderm induction media (Day 7 to Day 11). Table S1E. The formulation of parathyroid induction media (Day 12 to Day 18). Table S1F. The formulation of normal calcium concentration parathyroid induction media. Table S1G. The formulation of 500 mL low calcium concentration serum‐free basal medium (SFBM‐Low Ca). Table S1H. The formulation of low calcium concentration parathyroid induction media. Table S1I. The formulation of hESC‐derived parathyroid organoid media. [file CPR-57-e13634-s005.docx]

**Supplementary Table 1A. The formulation of 500ml serum-free basal medium (SFBM)**

| Reagent | Volume |
| --- | --- |
| IMDM | 375 mL |
| Ham’s F12 | 112 mL |
| N2 supplement | 2.5 mL |
| B27 supplement, without retinoic acid | 5 mL |
| ascorbic acid (50 mg/ml stock solution) | 500 μL |
| diluted MTG (13 µl/ml stock solution) | 1.5 mL |
| bovine serum albumin (BSA) Fraction V, 7.5% solution | 3.33 mL |
| penicillin/streptomycin | 500 μL |

**Supplementary Table 1B. The formulation of Definitive Endoderm induction media (Day1-Day3)**

| Reagent | Final concentration (Day 1) | Final concentration  (Day 2) | Final concentration  (Day 3) |
| --- | --- | --- | --- |
| 1640(with glutamax，HEPES) | / | / | / |
| penicillin/streptomycin | 1x | 1x | 1x |
| Human Activin A | 100 ng/mL | 100 ng/mL | 100 ng/mL |
| CHIR99021 | 3 μmol/L | / | / |
| Fetal bovine serum | / | 0.2% | 2.0% |

**Supplementary Table 1C. The formulation of Anterior Foregut Endoderm induction media (Day4-Day6)**

| Reagent | Final concentration (Day4-Day6) |
| --- | --- |
| SFBM | / |
| Noggin | 200ng/mL |
| SB431542 | 10µM |

**Supplementary Table 1D. The formulation of Pharyngeal Endoderm induction media (Day7-Day11)**

| Reagent | Final concentration (Day7-Day11) |
| --- | --- |
| SFBM | / |
| FgF8b | 50ng/ml |
| SB431542 | 10µM |
| RA | 0.1μM |
| shh | 100ng/ml |
| BMP4 | 10ng/mL |

**Supplementary Table 1E. The formulation of parathyroid induction media (Day12-Day18)**

| Reagent | Final concentration (Day12-Day18) |
| --- | --- |
| SFBM | / |
| FgF8b | 50ng/ml |
| SB431542 | 10µM |
| RA | 0.1μM |
| shh | 100ng/ml |
| Noggin | 200ng/mL |

**Supplementary Table 1F. The formulation of Normal Calcium Concentration parathyroid induction media**

| Reagent | Final concentration |
| --- | --- |
| SFBM | / |
| FgF8b | 50ng/ml |
| SB431542 | 10µM |
| RA | 0.1μM |
| shh | 100ng/ml |
| Noggin | 200ng/mL |

**Supplementary Table 1G. The formulation of 500ml Low calcium concentration serum-free basal medium (SFBM****-Low Ca)**

| Reagent | Volume |
| --- | --- |
| Calcium-free Dulbecco's Modified Eagle Medium (GIBCO cat. no. 21068028) | 500 mL |
| Calcium Chloride Solution (1mol/L, sterile) | 500 μl |
| N2 supplement | 2.5 mL |
| B27 supplement, without retinoic acid | 5 mL |
| ascorbic acid (50 mg/ml stock solution) | 500 μL |
| diluted MTG (13 µl/ml stock solution) | 1.5 mL |
| bovine serum albumin (BSA) Fraction V, 7.5% solution | 3.33 mL |
| penicillin/streptomycin | 500 μL |

**Supplementary Table 1H. The formulation of Low Calcium Concentration parathyroid induction media**

| Reagent | Final concentration |
| --- | --- |
| SFBM-Low Ca | / |
| FgF8b | 50ng/ml |
| SB431542 | 10µM |
| RA | 0.1μM |
| shh | 100ng/ml |
| Noggin | 200ng/mL |

**Supplementary Table 1I. The formulation of** **hESC-derived parathyroid organoid media**

| Reagent | Final concentration |
| --- | --- |
| SFBM | / |
| Noggin | 100ng/ml |
| R-Spondin-1 | 250ng/ml |
| CHIR99021 | 3μM |
| EGF | 50ng/ml |
| FgF8b | 50ng/ml |
| SB431542 | 10μM |
| RA | 0.1μM |
| shh | 100ng/ml |
| Y27632 | 10μM |
