# Supplementary material for: Directed differentiation of human embryonic stem cells into parathyroid cells and establishment of parathyroid organoids
Source: Cell Prolif. 2024 Mar 18;57(8):e13634. doi: 10.1111/cpr.13634 (PMC11294423; doi:10.1111/cpr.13634)
Supplement: Supplementary file 14 — Table S2. Details of the antibody and reagents used in this study. Table S2A. Antibody details and dilution ratios. Table S2B. Details of the reagents used in this study. [file CPR-57-e13634-s013.docx]

**Supplementary Table 2A. Antibody details and Dilution ratios.**

| Antibody | Company | Cat No. | Dilution ratio |
| --- | --- | --- | --- |
| PTH | Novus | NB100-65676 | 1:100 |
| CaSR | Novus | NB120-19347 | 1:100 |
| CHGA | Invitrogen | MA5-13096 | 1:100 |
| SOX17 | Abcam | ab84990 | 1:50 |
| FOXA2 | Abcam | ab108422 | 1:300 |
| SOX2 | R&D Systems | MAB2018-SP | 1:50 |
| CDX2 | Abcam | ab76541 | 1:500 |
| EYA1 | Atlas Antibodies | HPA028917 | 1:50 |
| HOXA3 | Atlas Antibodies | HPA029157 | 1:100 |
| TBX1 | Abcam | ab18530 | 1:100 |
| OCT4 | Abcam | ab19857 | 1 µg/ml |
| GCM2 | Abcam | ab201170 | 1:200 |
| GAPDH | Proteintech | 60004-1 | 1:5000 |
| Donkey anti-Mouse IgG (H+L) Highly Cross-Adsorbed Secondary Antibody, Alexa Fluor 594 | Invitrogen | A-21203 | 1:1000 |
| Donkey anti-Mouse IgG (H+L) Highly Cross-Adsorbed Secondary Antibody, Alexa Fluor 488, | Invitrogen | A-21202 | 1:1000 |
| Donkey anti-Rabbit IgG (H+L) Highly Cross-Adsorbed Secondary Antibody, Alexa Fluor 594 | Invitrogen | A-21207 | 1:1000 |
| Donkey anti-Rabbit IgG (H+L) Highly Cross-Adsorbed Secondary Antibody, Alexa Fluor 488 | Invitrogen | A-21206 | 1:1000 |
| HRP Goat anti Rabbit IgG (H+L) | Antgene | ANT020 | 1:3000 |
| HRP Goat anti Mouse IgG (H+L) | Antgene | ANT019 | 1:3000 |

**Supplementary Table 2B. Details of the Reagents used** **in this study**

| Reagent | Company | Cat. No. |
| --- | --- | --- |
| mTeSR Plus medium | StemCell Technologies | cat. no. 100-0276 |
| Iscove’s Modified Dulbecco’s Medium | Thermo Fisher Scientific | cat. no. 12440053 |
| 1640 medium | Thermo Fisher Scientific | cat. no. 61870-036 |
| Ham’s F12 medium | Thermo Fisher Scientific | cat. no. 11765054 |
| penicillin/streptomycin | Solarbio | cat. no. P1400 |
| DMEM/F12 | Thermo Fisher Scientific | cat. no. 11330-057 |
| N2 supplement | Invitrogen | cat. no. 17502-048 |
| B27 supplement | Invitrogen | cat. no. 12587-010 |
| BSA 7.5% stock (7.5 g/100 ml) | Solarbio | cat. no. H1130 |
| GlutaMAX | Thermo Fisher Scientific | cat. no. 35050-061 |
| 1-Thioglycerol (MTG) | Sigma-Aldrich | cat. no. M6145 |
| L-Ascorbic acid (L-AA) | Sigma-Aldrich | cat. no. A4544 |
| Activin A | Peprotech | cat. no. 120-14 |
| CHIR99021 | Stemgent | cat. no. 04-0004 |
| Noggin | Peprotech | cat. no. 120-10C |
| SB431542 | Selleck | cat. no. S1067 |
| FgF8b | Peprotech | cat. no. 100-25 |
| shh | Peprotech | cat. no. 100-45 |
| R-Spondin-1 | Peprotech | cat. no. 120-38 |
| EGF | Peprotech | cat. no. AF-100-15 |
| BMP4 | R&D Systems | cat. no. 314-BP-050 |
| Retinoic acid (RA) | Sigma-Aldrich | cat. no. R2625 |
| Y-27632 | StemCell Technologies | cat. no. 72302 |
| 0.05% Trypsin-EDTA | Thermo Fisher Scientific | cat. no. 25-300-062 |
| Accutase | StemCell Technologies | cat. no. 07920 |
| Growth factor-reduced Matrigel | Corning | cat. no. 356231 |
| Human ESC-qualified Matrigel | Corning | cat. no. 354277 |
| Paraformaldehyde | Servicebio | cat. no. G1101 |
| Dulbecco’s PBS | Thermo Fisher Scientific | cat. no. 14190144 |
| Dimethyl sulfoxide (DMSO) | Sigma-Aldrich | cat. no. D2650 |
| fetal bovine serum (FBS) | Thermo Fisher Scientific | cat. no. 16000-044 |
| Tris-EDTA antigen-repair solution | Servicebio | cat. no. G1206 |
| Donkey serum | Solarbio | cat. no. SL050 |
| Triton X-100 | Biofroxx | cat. no. 1139ML100 |
| DAPI | Servicebio | cat. no. G1012 |
| Fluoromount-G | SouthernBiotech | cat. no. 0100-01 |
| cell climbing slices | WHB scientific | cat. no. WHB-24-CS |
| Adhesion microscope slides | CITOTEST | cat. no. 188105 |
| microscope cover glass | CITOTEST | cat. no. 10212450C |
