# Supplementary material for: Directed differentiation of human embryonic stem cells into parathyroid cells and establishment of parathyroid organoids
Source: Cell Prolif. 2024 Mar 18;57(8):e13634. doi: 10.1111/cpr.13634 (PMC11294423; doi:10.1111/cpr.13634)
Supplement: Supplementary file 15 — Table S3. Details of the primers used in this study. [file CPR-57-e13634-s001.docx]

**Supplementary Table 3. Details of the Primers used in this study**

| **Gene** | **Sequence** |
| --- | --- |
| CDX2-F | GACGTGAGCATGTACCCTAGC |
| CDX2-R | GCGTAGCCATTCCAGTCCT |
| SOX2-F | TGGACAGTTACGCGCACAT |
| SOX2-R | CGAGTAGGACATGCTGTAGGT |
| MIXL1-F | GGCGTCAGAGTGGGAAATCC |
| MIXL1-R | GGCAGGCAGTTCACATCTACC |
| FOXA2-F | GGAGCAGCTACTATGCAGAGC |
| FOXA2-R | CGTGTTCATGCCGTTCATCC |
| SOX17-F | GTGGACCGCACGGAATTTG |
| SOX17-R | GGAGATTCACACCGGAGTCA |
| NKX2-1-F | AGCACACGACTCCGTTCTC |
| NKX2-1-R | GCCCACTTTCTTGTAGCTTTCC |
| CXCR4-F | ACTACACCGAGGAAATGGGCT |
| CXCR4-R | CCCACAATGCCAGTTAAGAAGA |
| HOXA3-1F | ATGCAAAAAGCGACCTACTACG |
| HOXA3-1R | TACGGCTGCTGATTGGCATTA |
| HOXA3-2F | GCCAGCCCTCTTTGGTCTAAC |
| HOXA3-2R | GCCGGTAAGGTCCGTGTAG |
| EYA1-1F | CACCACAGATTTACCCTTCCAAC |
| EYA1-1R | GTACGTGGCATAGGCTGTAGC |
| EYA1-2F | GGACTATCCGTCTTATCCCAGT |
| EYA1-2R | GCTGCTGGTCATATAATGTGCTG |
| PAX9-1F | GGAGGAGTGTTCGTGAACGG |
| PAX9-1R | CGGCTGATGTCACACGGTC |
| PAX9-2F | GGCGTGTGCGACAAGTACA |
| PAX9-2R | GGGCCAAGTTGCCGATCTT |
| Six1-F | CTGCCGTCGTTTGGCTTTAC |
| Six1-R | GCTCTCGTTCTTGTGCAGGT |
| Pax1-F | CACACTCGGTCAGCAACATC |
| Pax1-R | CATCTTGGGAGAGTAAGCGGT |
| TBX1-1F | TAGCGAGAAATATGCCGAGGA |
| TBX1-1R | CGTGATCCGATGGTTCTGGT |
| TBX1-2F | GTCTATGTGGACCCACGCAA |
| TBX1-2R | CTGCGTGATCCGATGGTTCT |
| GCM2-F | ACAGCGGATACCCCGTAAC |
| GCM2-R | TGATGGCGCTTCTTCTAGCTT |
| CaSR-1F | CCAACTTGACGCTGGGATACA |
| CaSR-1R | CAGCAATCGTAGAGGGAATGTG |
| CaSR-2F | CCCTCTACGATTGCTGTGGTG |
| CaSR-2R | AGGAGGCATAACTGACCTGGG |
| PTH-1F | GCGTAAGAAGCTGCAGGATG |
| PTH-1R | TGGCTCTCAACCAAGACATTG |
| PTH-2F | GAGTAGAATGGCTGCGTAAGAAG |
| PTH-2R | TTCATGGCTCTCAACCAAGAC |
| Gata3-F | GCCCCTCATTAAGCCCAAG |
| Gata3-R | TTGTGGTGGTCTGACAGTTCG |
| GATA4-1F | TAGCCCCACAGTTGACACAC |
| GATA4-1R | GTCCTGCACAGCCTGCC |
| GATA4-2F | CGACACCCCAATCTCGATATG |
| GATA4-2R | GTTGCACAGATAGTGACCCGT |
| MAFB-F | TCAAGTTCGACGTGAAGAAGG |
| MAFB-R | GTTCATCTGCTGGTAGTTGCT |
| CHGA-F | TAAAGGGGATACCGAGGTGATG |
| CHGA-R | TCGGAGTGTCTCAAAACATTCC |
| FOXN1-1F | CTGCTCGTCATTTGTGTCCGA |
| FOXN1-1R | AGCCAAAGCCAGGATACTTGT |
| FOXN1-2F | CCCTTCCACCCGTACAAGC |
| FOXN1-2R | CCTGGGGTCTTAAAGGAGTGTC |
| GAPDH-F | GGAGCGAGATCCCTCCAAAAT |
| GAPDH-R | GGCTGTTGTCATACTTCTCATGG |

F=Forward Primer, R=Reverse Primer
